# Identifying and developing effective post‐2020 conservation bridging leaders

**DOI:** 10.1111/cobi.13980

**Published:** 2022-10-06

**Authors:** Wayne Stanley Rice

**Affiliations:** ^1^ Amsterdam Institute for Social Science Research (AISSR) University of Amsterdam Amsterdam The Netherlands; ^2^ Department of Environmental and Geographical Sciences University of Cape Town Cape Town South Africa

**Keywords:** collaborative governance, community‐based conservation, integrative leadership, leadership characteristics, leadership competencies, características de liderazgo, competencias de liderazgo, conservación basada en la comunidad, liderazgo integrador, gestión colaborativa, 合作管理, 基于社区的保护, 综合领导力, 领导特征, 领导能力

## Abstract

Effective bridging leaders interact within and outside their group to facilitate collaboration required in multistakeholder contexts. This is particularly crucial to community‐based conservation interventions that strive to achieve both ecological and social objectives by actively engaging or devolving decision‐making and management authority to local communities. Although a viable approach in many contexts, achieving “unprecedented collaboration” called for by the Post‐2020 Global Biodiversity Framework in community‐based conservation is problematic given the multiple and diverse actors affecting and affected by these interventions. Therefore, effective leadership becomes crucial to implementing necessary strategies to engage actors and resolve conflict inclusively. Yet, weak leadership commonly constrains these interventions. I reviewed relevant literature and devised a framework of effective bridging leadership characteristics. I then used this framework to appraise bridging leadership in two African coastal‐marine community‐based conservation cases. I employed social network analysis and semistructured and group interviews in the two cases. Several local leaders emerged as key (potential) bridging leaders. Furthermore, I found that effective bridging leaders require not only legal recognition but also perceived legitimacy resulting from building trust with other actors. Additionally, the inclusive collaboration required multiple sources of emotionally intelligent bridging leaders with the integrity, humility, empathy, and cultural awareness necessary to mitigate elite capture, effectively communicate, and empower and provide support to others. Because emotional intelligence in conservation leadership remains a knowledge gap, particularly in community‐based conservation research, insights from this study should be useful to diverse conservation actors.

## Resumen

Los líderes vinculantes efectivos interactúan dentro y fuera de su grupo para facilitar la colaboración requerida en contextos con múltiples actores. Esto es crucial para las intervenciones de conservación basada en la comunidad que buscan alcanzar objetivos ecológicos y sociales mediante la participación y transferencia de la autoridad gestora y la toma de decisiones a la comunidad local. Aunque es una estrategia viable, es problemático lograr la “colaboración sin precedentes” en la conservación basada en la comunidad que exige el Marco Mundial de Biodiversidad Post‐2020 debido a los múltiples y diversos actores que afectan y han sido afectados por estas intervenciones. Por lo tanto, el liderazgo efectivo se vuelve crucial para la implementación de las estrategias necesarias para involucrar a los actores y resolver los conflictos de manera inclusiva. Sin embargo, es común que el liderazgo deficiente limite estas intervenciones. Revisé la literatura relevante y diseñé un marco de trabajo de las características del liderazgo vinculante efectivo. Después usé este marco para valorar el liderazgo vinculante en dos casos de conservación costera‐marina basada en la comunidad en África. Analicé las redes sociales y realicé entrevistas grupales y semiestructuradas en ambos casos. Además, descubrí que los líderes vinculantes efectivos requieren no sólo de un reconocimiento legal, sino también de la legitimidad resultante de la construcción de la confianza con otros actores. Asimismo, la colaboración inclusiva requirió de líderes vinculantes de múltiples orígenes con inteligencia emocional y con la integridad, humildad, empatía y conciencia cultural para mitigar la captura elite, comunicar efectivamente y empoderar y proporcionarles apoyo a los demás. Ya que la inteligencia emocional todavía es un vacío de conocimiento en el liderazgo de la conservación, particularmente en la investigación sobre la conservación basada en la comunidad, el conocimiento que brinda este estudio debería ser útil para diversificar a los actores de la conservación.

## 【摘要】

有影响力的桥接领导者可以在团体内外相互交流, 以促进多方利益相关者的合作。这对以社区为基础的保护干预措施尤为关键, 这些干预措施通过积极地将决策和管理权力分享或移交到当地社区, 来努力实现生态和社会目标。虽然在许多情况下这都是一种可行的方法, 但考虑到影响这些干预措施和受其影响的行为者的多样性, 在基于社区的保护中实现2020年后全球生物多样性框架所要求的“前所未有的合作”仍面临挑战。因此, 有效领导成为了实施必要战略、促进行为者参与、以包容性的方式解决冲突的关键。然而, 薄弱的领导力通常会限制这些干预措施。本研究回顾了相关文献, 并设计了一个有效的桥接领导特征框架。接下来, 研究者使用这个框架对两个非洲沿海海洋社区保护案例中的桥接领导力进行了评估。在这两个案例中, 研究者采用社会网络分析以及半结构化访谈和小组访谈的方法, 发现一些当地领导者是关键(潜在)的桥接领导者。此外, 研究发现有效的桥接领导者不仅需要法律上的认可, 还需要通过与其他行为者建立信任而获得他人认可的正当性。此外, 包容性合作需要多种来源的高情商桥接领导者, 他们应正直、谦逊、有同理心和文化意识, 以减少精英俘获、实现有效地沟通, 并赋予他人权力和提供支持。由于保护领导力中的情商仍然是一项知识空缺, 在基于社区的保护领域尤为如此, 因此本研究的见解应有助于不同的保护行为者。**【翻译:胡怡思;审校:聂永刚】**

## INTRODUCTION

Scholars and practitioners have increasingly called for an improved multidisciplinary and contextual understanding of leadership to better address complex, contemporary societal challenges (e.g., Osland, et al., 2013; Urton & Murray, 2021). Notwithstanding progress to date, understanding what constitutes effective leadership in conservation remains less well developed relative to other fields (Englefield et al., 2019; Webb et al., 2021). In particular, a more in‐depth understanding of leadership complexities in community‐based conservation (CBC) interventions is increasingly demanded (e.g., Alexander et al., 2018; Steenbergen & Warren, 2018).

The CBC approach strives to actively engage or devolve decision‐making and management authority over natural resources to Indigenous peoples and local communities (IPLCs), which is increasingly recognized as key to conservation success (e.g., Corrigan et al., 2018; Dawson et al., 2021; Walters et al., 2021). Not surprisingly, the Post‐2020 Global Biodiversity Framework (Post‐2020 GBF) specifically calls for “the full and effective participation of indigenous peoples and local communities” (CBD, 2020: 7) to reduce biodiversity loss and sustainably meet people's needs. Like the Aichi 2020 Targets before, the Post‐2020 GBF specifically recognizes the contributions of Indigenous peoples and community‐conserved territories and areas (ICCAs) (CBD, 2020: 9). Sajeva et al. (2019: 5) define ICCAs as “territories and areas governed, managed and conserved by custodian indigenous peoples and local communities.” Yet, governance arrangements in ICCAs vary greatly in the degrees of engagement or decision‐making authority of IPLCs and the levels of nested partner support (Sajeva et al., 2019).

Although CBC offers a viable approach in many contexts, achieving desired collaboration is often problematic given the multiple and diverse actors affecting and affected by these interventions and necessitates strategies to engage all actors and resolve conflict inclusively (Armitage et al., 2020; Rice, 2022). Effective leadership is central to this endeavor.

Leadership can be framed as it relates to a person, the position held, and as a process or result (Case et al., 2015). I explored the characteristics of effective CBC leaders (i.e., the person) because this remains a specific knowledge gap. However, a leader's effectiveness will influence and be influenced by the process and their ability to produce results. Not surprisingly, leadership characteristics are insufficiently understood because the complexity of what constitutes effective leadership requires an understanding of not only the leader but also how leadership is socially constructed through relations (Jansson et al., 2021). I refer to leadership characteristics throughout as the overarching values, competencies, skills, and behaviors that enable a leader to positively influence others to contribute toward a shared objective to conserve or restore a social‐ecological system (Englefield et al., 2019; Webb et al., 2021).

Ospina and Foldy (2010: 303) describe bridging leadership as “the leadership work that connects different perspectives … to facilitate collaboration, but in ways that value those differences and maintain them for the sake of the broader vision.” Therefore, bridging leadership considers a leader's ability to effectively develop relational capital within diverse multistakeholder groups to promote required collaboration, resolve conflict, and achieve greater collective action (Ospina & Foldy, 2010; Jansson et al., 2021). Consequently, bridging leadership is central to CBC interventions that require collaboration among IPLCs, governmental and nongovernmental partners, and others. I define a *CBC bridging leader* as an influential actor who is well‐positioned to effectively interact with other actors within and outside their group to facilitate governance processes in a CBC intervention (Crona et al., 2017; Barnes et al., 2019).

Extensive studies of diverse coastal‐marine CBC interventions emphasize that bridging leaders (also known as brokers, opinion leaders, or institutional entrepreneurs) are crucial to achieving desired ecological and social outcomes (e.g., Lyons & Cavaye, 2016; Crona et al., 2017; Steenbergen & Warren, 2018). More specific to the two cases discussed subsequently, African coastal‐marine CBC studies frequently acknowledge a lack of recognition and ineffective use of bridging leaders as a missed opportunity for success (e.g., Mbaru & Barnes, 2017; Barnes et al., 2019). Consequently, I reviewed the relevant literature to determine a framework of effective CBC bridging leadership characteristics, which I then appraised using mixed methods in two African coastal‐marine ICCAs. Finally, I devised recommendations for identifying and developing effective CBC bridging leaders. In doing so, I strove to contribute to the aforementioned knowledge gap, which requires a more in‐depth understanding of leadership complexities in CBC interventions.

## CHARACTERISTICS OF EFFECTIVE BRIDGING LEADERS

### Broader leadership literature

Numerous leadership theories and approaches propose effective leadership characteristics (Northouse, 2016). First, and notwithstanding their process‐ and context‐based limitations and their leader‐centric nature, the leadership traits and skills approaches provide a useful perspective that emphasizes that effective leaders require self‐confidence, integrity, motivation, and cognitive abilities: specifically, problem‐solving and social skills (Appendix S1).

I reviewed several values‐based leadership theories, which included servant leadership, authentic leadership, ethical leadership, and spiritual leadership (Appendix S1). These approaches focus on a leader's ability to motivate and empower others. Although criticized for being overly optimistic, lacking in causal validity, and requiring more rigorous research, they do offer valuable insights for CBC bridging leaders because they emphasize how a leader should target their actions toward others (Alvesson & Einola, 2019). This also links to emotional intelligence, a well‐recognized topic of importance and debate in the leadership literature (Dasborough et al., 2021). An emotionally intelligent leader possesses the ability to both identify and manage their own emotions and assess the emotional effect of their actions on others in the pursuit of intervention outcomes (Goleman, 2004; Dong et al., 2022).

Goleman (2004) refers to five components of emotional intelligence: self‐awareness, self‐regulation, self‐motivation, empathy, and social skill. Accordingly, these components influence how a leader makes choices and engages with others (Sousa & van Dierendonck, 2017). Self‐awareness and self‐regulation identify several overlapping leadership characteristics, including humility, integrity, and transparency (Goleman, 2004) (Appendix S1). Humility is an often overlooked aspect of leadership effectiveness, yet it is influential in a leader's interactions (Sousa & van Dierendonck, 2017). Furthermore, while the meaning of *humility* lacks consensus, integrity is generally understood as the consistency of a leader's words, values, and actions, which, like humility, assist in obtaining the support of others (Martin et al., 2013). Although a leader's integrity may manifest in contextually and culturally specific behaviors, it can be considered inclusive of honesty, fairness, transparency, and the role modeling of normatively desirable attitudes and behaviors (Martin et al., 2013). Similarly, a positive predictor of developing normatively desirable attitudes and behavior in others is a leader perceived by others to provide actual assistance or inspire feelings of attachment to the leader, group, or intervention (Jolly et al., 2020). Accordingly, the final two emotional intelligence components of empathy and social skills emphasize a leader's ability to understand, interact with, and develop and empower others (Goleman, 2004) (Appendix S1). Last, emotionally intelligent leaders possess optimism, which inspires others (Norman et al., 2010).

### Conservation practitioner literature

To build on the above theoretical foundation, I reviewed the conservation practitioner literature. Summarized, the common characteristics are the need to possess legitimacy and the ability (and willingness) of leaders to develop support networks, build trust, facilitate engagement, establish a shared vision, communicate effectively, motivate others, and manage conflicts (Appendix S2). Straka et al. (2018) specifically emphasized the prior lack of consideration and the increasing need for conservation leaders to possess greater cultural awareness. Black (2021: 321–322) recently emphasized the importance of conservation leaders “authentically engaging” with others and placing “purpose before ego” (i.e., humility).

### Community‐based conservation literature

**FIGURE 1:**
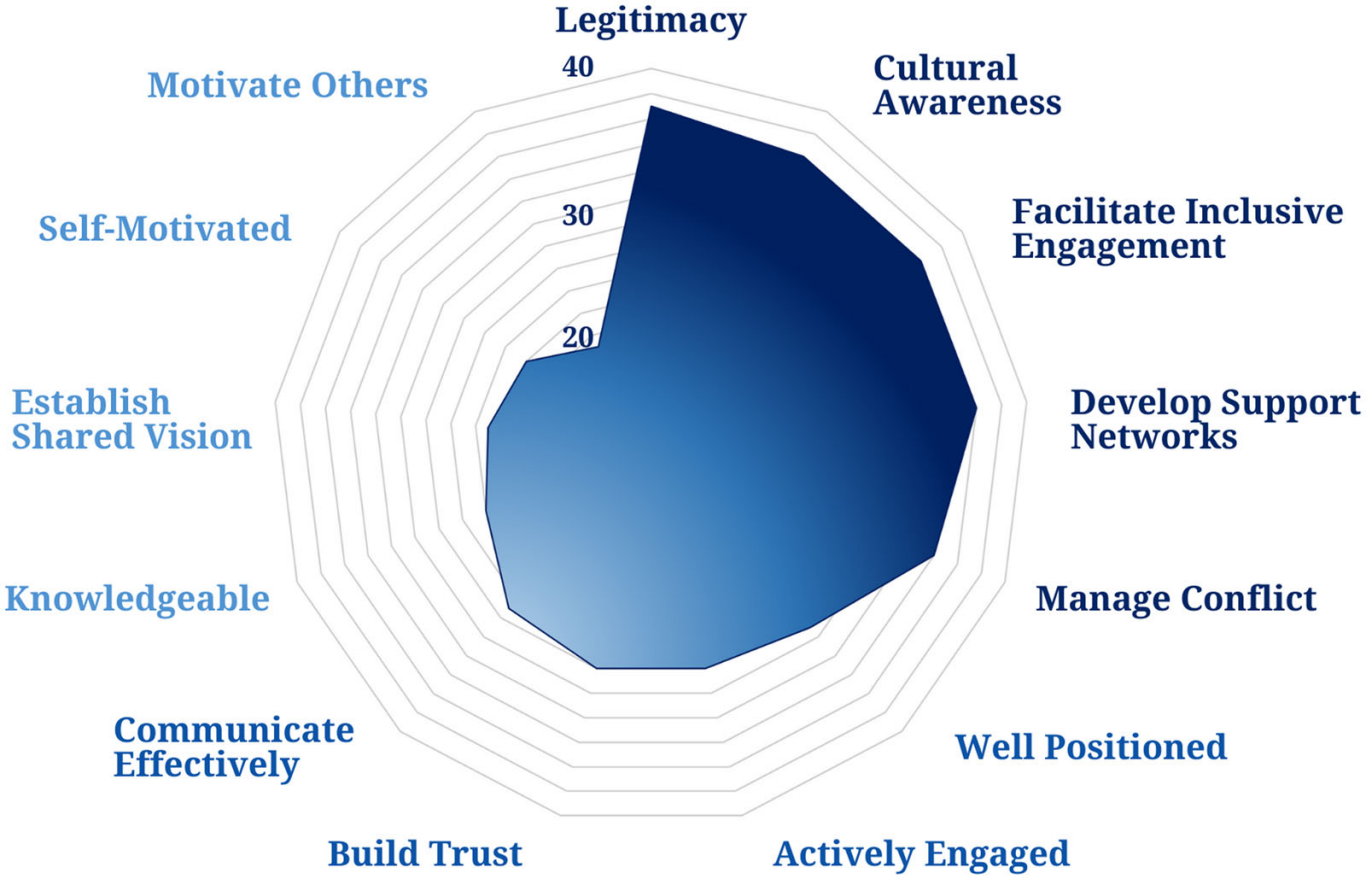
The frequency of mention of key identified effective bridging leadership characteristics that emerged from the 54 reviewed community‐based conservation studies. Leadership characteristics decrease in frequency (light blue) clockwise from *legitimacy*.

The aforementioned conservation leadership characteristics guided my review of the CBC‐specific literature, in which I sought studies on common effective leadership characteristics, particularly those related to coastal‐marine interventions in developing nations over the last two decades (details of the review methods are in Appendix S3). Although process and outcome understandings of leadership frequently appear in the CBC literature, research addressing specific leadership characteristics is scarce. Nevertheless, the 54 CBC studies I reviewed identified several common characteristics of effective CBC leaders (Figure 1) (list of included studies in Appendix S3).

The literature I reviewed emphasized the need for CBC bridging leaders to possess legitimacy (Figure 1). More specifically, while legitimacy is assisted by legal recognition, the literature emphasized that a CBC leader's legitimacy appears more contingent on their ability to gain local respect and recognition by being knowledgeable and transparent and by building trust and communicating effectively. Furthermore, this perceived legitimacy appeared crucial to a CBC leader's ability to motivate others, establish a shared vision, develop support networks, manage conflicts, and, ultimately, facilitate inclusive engagement. These findings relative to a leader's legitimacy mirror those of a recent systematic qualitative review (Golebie et al., 2022). Moreover, as Straka et al. (2018) found, cultural awareness emerged central to multiple aspects of effective CBC leadership, most notably a leader's ability to motivate others, establish a shared vision, and facilitate inclusive engagement, particularly in culturally diverse communities (i.e., a common CBC contextual factor). Additional commonly identified characteristics were the need for leaders to be well positioned, self‐motivated, and actively engaged over the long term, which appeared central to a CBC leader's ability to motivate others and facilitate inclusive engagement (see Appendix S3).

### Consolidated framework of effective community‐based conservation bridging leadership characteristics

**FIGURE 2:**
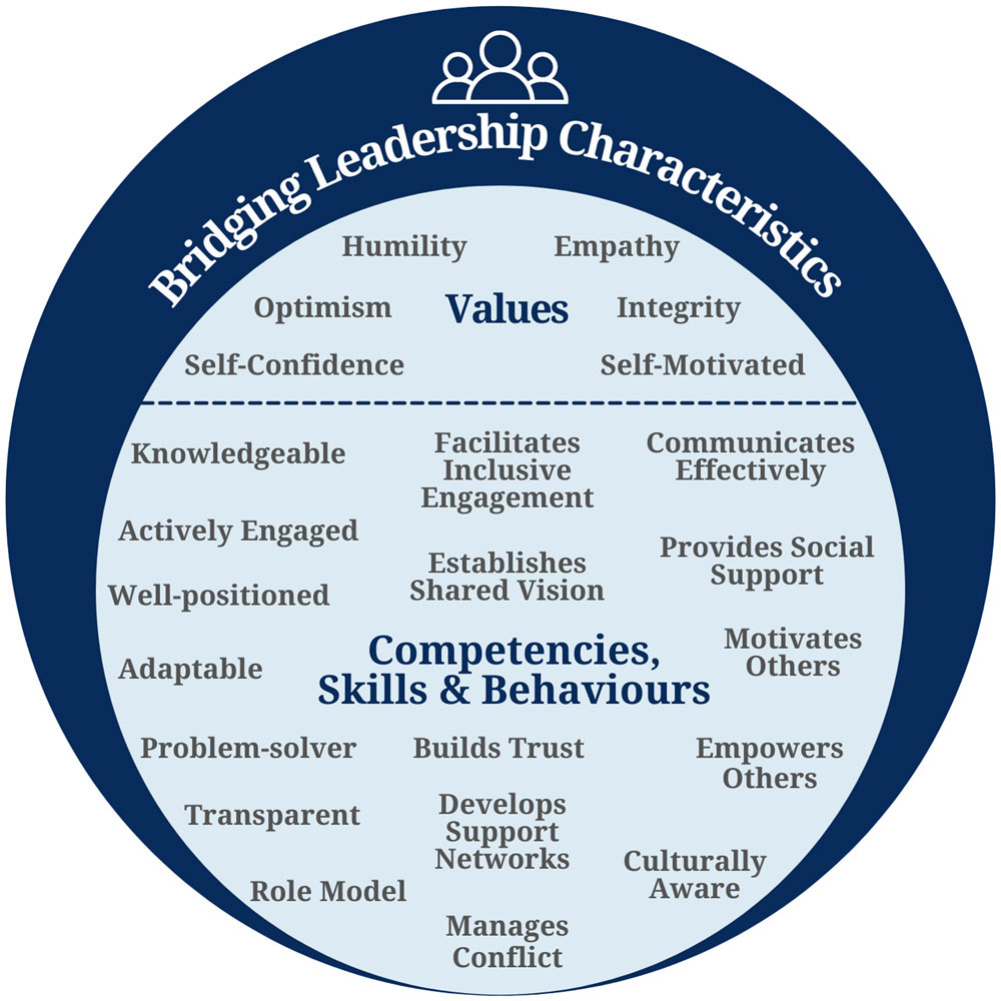
A framework of effective community‐based conservation bridging leadership characteristics.

In addition to those characteristics identified in the aforementioned CBC literature review (Figure 1) and based on the other literature I reviewed, I identified several additional characteristics of specific relevance to effective CBC bridging leaders, most notably, characteristics encapsulating a leader's emotional intelligence. More specifically, several value characteristics of effective leaders predominated the leadership literature that appear especially relevant to CBC interventions: empathy, integrity, humility, and optimism. Several competencies, skills, and behaviors of effective leaders were also identified from this literature, including a leader's ability to be a problem solver, be adaptable, provide social support, communicate effectively, and empower others. Consequently, I devised a framework of common, effective CBC bridging leadership characteristics (Figure 2). Although this framework does not provide an exhaustive list of characteristics of an effective leader and each characteristic was not extensively critiqued, it provides a useful guide for exploring bridging leadership in the two selected case studies.

## METHODS

### Case studies

**FIGURE 3:**
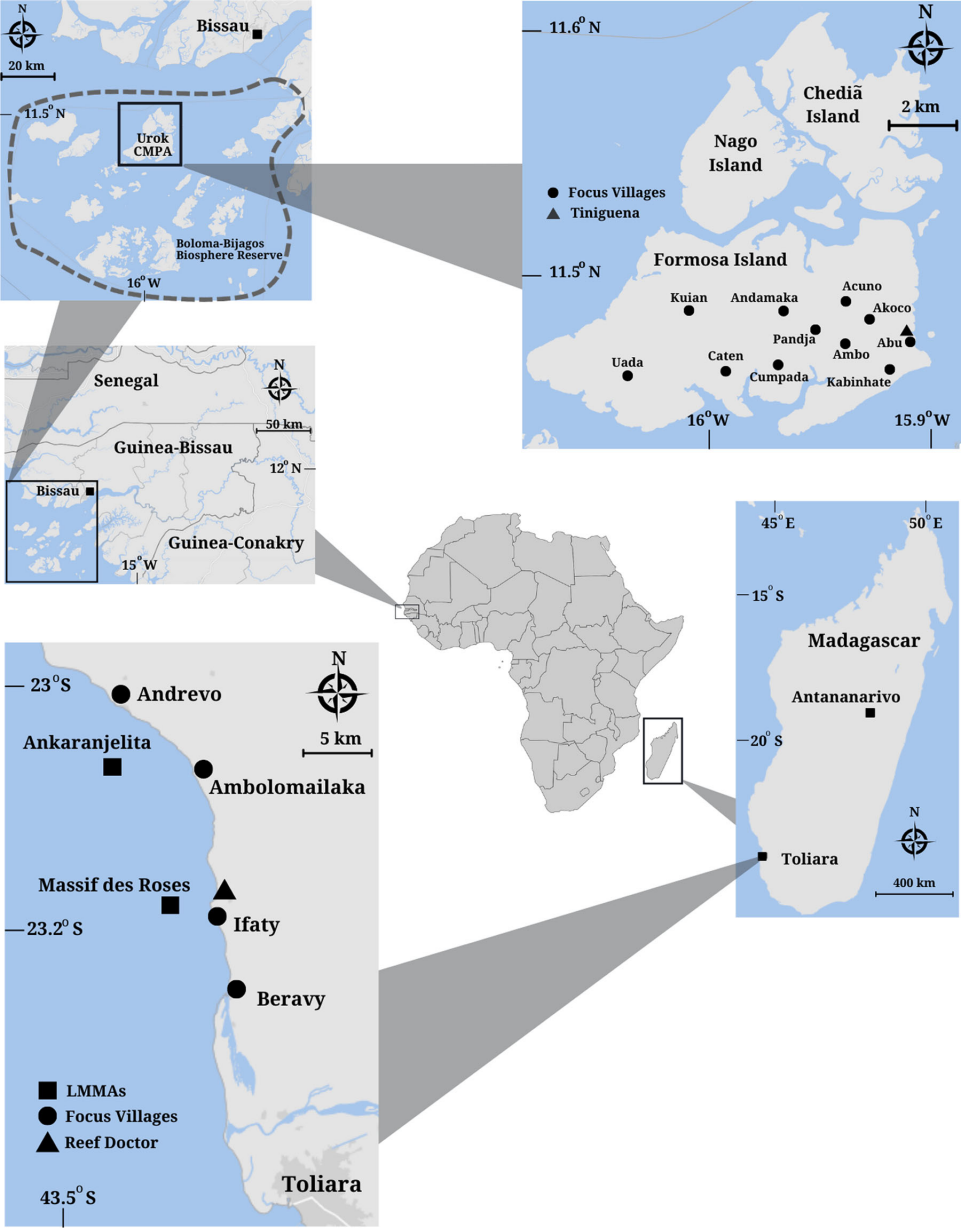
Locations of the Bay of Ranobe, Madagascar, and the Urok Islands, Guinea‐Bissau and of the conservation areas, villages where data were collected, and key partners (i.e., Reef Doctor and Tiniguena).

I appraised the framework (Figure 2) through case studies of CBC interventions in the Bay of Ranobe, Madagascar, and Urok Islands, Guinea‐Bissau (Figure 3). I used a case study approach because it can capture the complexities of real‐world, lived experiences of conservation stakeholders (Moon et al., 2019).

The Bay of Ranobe (Figure 3) is a coastal lagoon in southwest Madagascar that is rich in marine biodiversity (Belle et al., 2009). The bay is home to approximately 8000 fishers from 13 villages, the majority of which identify as Vezo, a people for whom the sea is culturally important and a primary livelihood source (Cripps & Gardner, 2016). Vezo cultural practices, such as *fady* (i.e., daily taboos), and *dina* (i.e., the local system of establishing rules based on sociocultural norms ratified by village presidents), still affect daily life, including natural resource use (Cripps & Gardner, 2016; Gardner et al., 2020). Nevertheless, the area has experienced increased coastal migration of traditionally inland peoples (e.g., the Masikoro [i.e., crop and livestock farmers]) (Cripps & Gardner, 2016). In April 2007, the community‐led association FIMIHARA was formed to establish and manage a locally managed marine area (LMMA) (Belle et al., 2009). The association comprises fisher representatives, Reef Doctor (a locally based British nongovernmental organization [NGO] working in the bay since 2002), and other NGO, governmental, and private sector partners (Belle et al., 2009). In 2007 and 2009, respectively, the Massif des Roses and Ankaranjelita LMMAs were established by a dina (Belle et al., 2009; Reef Doctor, 2012) (Figure 3). Both LMMAs have since received full legal recognition. More recently, the Vatosoa Marine Reserve was declared in January 2019 (Reef Doctor, 2019).

The Urok Islands are part of the Bijagós Archipelago, located off the coast of Guinea‐Bissau, in West Africa (Figure 3). The archipelago possesses high levels of biodiversity and is recognized as a key area for migratory birds (Campredon & Catry, 2018). Furthermore, most of Guinea‐Bissau's fishing activity takes place in the archipelago, and the area accounts for a significant portion of regional landings, including high levels of illegal, unreported, and unregulated fishing (Okafor‐Yarwood, 2019). The islands are home to approximately 3000 inhabitants, approximately 90% of whom identify as the traditionally animist Bijagó people, for whom fish is the primary protein source and shellfish are central to cultural ceremonies (Brenier et al., 2009). In July 2005, the state legally recognized the Urok Islands Community‐Managed Marine Protected Area (CMPA), the country's first and only CMPA to date (Brenier et al., 2009; Tiniguena, 2022). The CMPA comprises the three Urok Islands of Formosa, Nago, and Chediã and is part of the Bolama and Bijagós Archipelago Biosphere Reserve (Figure 3). The CMPA is managed by the Urok Management Committee (UMC), which includes fisher representatives, local customary authorities, Tiniguena (i.e., a national NGO), the Institute for Biodiversity and Protected Areas (i.e., the parastatal conservation agency and central CMPA oversight body), and additional state partners (Brenier et al., 2009). In 2019, the CMPA was recognized as an Equator Initiative prizewinner (Equator Initiative, 2019).

### Data collection and interpretation

I used mixed methods and data triangulation, which are increasingly recognized as approaches that improve understanding of conservation management (e.g., Kinnebrew et al., 2021). Fieldwork was conducted for approximately 2 months in each case (Madagascar from October to November 2016 and in Guinea‐Bissau from February to March 2018). I employed a questionnaire to conduct social network analysis, and collected qualitative data from semistructured interviews with 162 community respondents, inclusive of local representatives (i.e., 82 from Bay of Ranobe and 80 from Urok Islands) (Table 1).

**TABLE 1:** Details of the protocol in case studies of effective bridging leadership characteristics in community‐based conservation interventions

Case study	Number of respondents interviewed	Number of participants in informal group interviews	Participant observation
Bay of Ranobe, Madagascar	community respondents (82): by gender: male (46) female (36) local representatives: FIMIHARA (7) village presidents (3) by village: Ifaty (22) Beravy (20) Ambolomailaka (20) Andrevo (20) partner organization respondents (25): NGO (16) state (7) academic (2)	group 1 (October 17, 2016): male fishers on the beach near Ifaty village (7) group 2 (October 18, 2016): male elders in Ifaty village (4) group 3 (October 23, 2016): male fishers in Beravy village (8) group 4 (October 26, 2016): male fishers during a fishing trip near Ifaty village (2) group 5 (November 2, 2016): observed and informally interviewed a group of women and children during reef gleaning activities near Ifaty village (18) group 6 (November 4, 2016): male fishers during a fishing trip near Ifaty village (2) group 7 (November 8, 2016): women from the Masikoro tribe living in Ambolomailaka village (14) group 8 (November 8, 2016): male fishers in Ambolomailaka village (20) group 9 (November 12, 2016): male fishers on the beach near Andrevo village (5)	attended FIMIHARA monthly meeting held near Ifaty village (November 4, 2016) participated in a local aquaculture project near Ifaty village (November 6, 2016) accompanied fishers on two nearshore fishing trips near Ifaty village (October 26, 2016 and November 4, 2016)
Urok Islands, Guinea‐Bissau	community respondents (80): by gender: male (51) female (29) local representatives: customary Authorities (11) Urok Management committee (7) by village: Abu (11) Kabinhate (8) Akoco (9) Ambo (7) Acuno (8) Pandja (8) Andamaka (8) Cumpada (6) Kuian (5) Caten (4) Uada (6) partner organization respondents (14): NGO (10) atate (4)	group 1 ( March 8, 2018): informal community gathering of men and women in Kuian village (20) group 2 (March 12, 2018): informal community gathering of men and women in Cumpada village (14) group 3 (March 12, 2018): male fishers in Andamaka village (4) group 4 (March 22, 2018): men associated with bird monitoring project from Nago and Chediã islands (12) group 5 (March 22, 2018): women associated with shellfish monitoring project from Nago and Chediã Islands (13) group 6 (March 23, 2018): male fishers during a fishing trip near Abu village (4)	attended UMC monthly meeting in Abu village (February 12, 2018) attended a workshop for bird monitoring project in Abu village (March 22, 2018) attended a workshop for shellfish monitoring project in Abu village (March 22, 2018) accompanied fishers on a nearshore fishing trip near Abu village (February 23, 2018)

Community respondents were initially identified using convenience sampling and were subsequently purposively sampled to engage key local leaders and obtain a greater gender balance of respondents (Table 1). Focus villages were selected on the advice of local partner organizations and specifically included villages most actively involved in marine resource harvesting and villages located farther from the respective intervention's headquarters (Table 1). Unfortunately, due to logistical challenges encountered in the Urok Islands, I could not reach Nago and Chediã Islands, so fieldwork took place only on Formosa Island. However, Formosa is the most populous (i.e., approximately 80% of the Urok Islands’ population), and I was able to conduct two group interviews with residents of Nago and Chediã who visited Formosa (Table 1). The first group interview was conducted with 12 men associated with a local bird‐monitoring project, and the second was conducted with 13 women associated with a local shellfish‐monitoring project (Table 1). Although sample sizes in certain outlying villages on Formosa were low, due to the small populations of these villages (e.g., Caten has only eight households), and theoretical saturation was obtained among villages farther west of the intervention's headquarters in Abu, these data were considered sufficient and deemed crucial to capturing a more inclusive respondent group.

Social network analysis is a tool increasingly used to evaluate the nature of network structure and actors’ positions in conservation management (Mbaru & Barnes, 2017; Guerrero et al., 2020) and is particularly useful for examining leadership (Cullen‐Lester, et al., 2017). Accordingly, I employed a social relations and network appraisal questionnaire (SRNA) with the 162 community respondents introduced above (Table 1) (SRNA survey questions are in Appendix S4). The SRNA was used to identify (potential) bridging leaders, by asking respondents to state who they perceived to possess ultimate decision‐making power associated with their respective interventions; to assess effective communication, by asking respondents to identify sources of knowledge exchange; and to determine sources of social support. These latter two aspects are considered key to effective CBC governance (e.g., Crona et al., 2017; Barnes et al., 2019). I considered knowledge exchange from the perspective of both the community members who received knowledge related to CBC interventions (i.e., acquired knowledge) and those who shared their knowledge (i.e., spread knowledge). Sources of social support were assessed by asking respondents to identify those actors they deemed most approachable to interact with regarding their concerns related to natural resource access and use in their ICCAs.

The SRNA limited respondents to a maximum of three responses for each question, inclusive of individuals and organizations. Furthermore, other designations emerged from responses (e.g., “don't know” and “all stakeholders”). I recorded responses in two‐dimensional matrices in Microsoft Excel from which data sets and, subsequently, social network maps were developed using UCINET 6 Social Network Analysis software (Borgatti et al., 2002). Social network maps showed the degree of centrality (i.e., the number of connections possessed by the respondent to other actors or the number of responses for that actor), which is considered robust to (potential) missing data (Guerrero et al., 2020).

Qualitative approaches can provide a more nuanced and in‐depth understanding required of complex, contemporary conservation issues (Moon et al., 2019). Accordingly, I conducted semistructured interviews and informal group interviews and observed participants at multistakeholder meetings and during natural resource harvesting activities to supplement the SRNA and corroborate its results (Table 1). Individual interviews were transcribed, and detailed notes were taken during the informal group interviews and participant observations. I analyzed these data with an iterative and qualitative‐interpretive approach (Elliott & Timulak, 2021) and employed reflexive thematic analysis (Braun & Clarke, 2006). Although I acknowledge the potential for subjectivity with this approach, it offered a flexible way to gain a more nuanced understanding of leadership.

The face‐to‐face semistructured interviews were conducted with the same 162 community respondents described above and occurred directly after the SRNA. Thirty‐nine purposefully sampled key‐informant partner respondents were also interviewed (Table 1). Partner respondents included national and case‐specific organizations from state departments, NGOs, the private sector, and academic institutions (25 from Bay of Ranobe; 14 from Urok Islands) (Table 1). All interviews were conducted with the assistance of local translators who were university graduates fluent in diverse local dialects. The SRNA and interview questions were translated prior to data collection by the translators. Although I acknowledge the potential limitations of using translators, their knowledge associated with subtleties in language and culture proved invaluable to the interview process.

Community interviews lasted from 20 to 40 min, and partner interviews were from 40 to 60 min. All interviews employed a conversational technique that explained or rephrased questions to promote respondent understanding and response accuracy (Conrad & Schober, 2020). Sample sizes were not determined prior to data collection; rather, they were based on reaching perceived theoretical saturation, which was deemed appropriate given the research topic and approach (Sim et al., 2018). The interview questions (Appendix S4) focused on perceived challenges experienced in planning, implementing, and ongoing governance of the respective ICCAs, in particular, associated with community representation, and community perceived relations with identified leaders. Interview questions were revised prior to fieldwork based on a pilot study conducted in 2015 with 20 respondents involved in conservation management in South Africa.

Informal group interviews and participant observation further supplemented and corroborated the above data, and in particular, assisted in capturing insights from minority and commonly marginalized groups, most notably women (Table 1). Informal group interviews involved a brief introduction to the research objectives and an open‐ended discussion of roles and challenges related to the interventions, in accordance with individual interviews. Finally, participant observation in each case included accompanying community members on multiple marine resource harvesting trips and attendance at several local village and multistakeholder meetings, including those of both FIMIHARA and UMC (Table 1). These two methods offered valuable opportunities to provide feedback and clarify emerging findings with a subset of case respondents.

Ethical clearance was obtained from the University of Cape Town research ethics committee prior to fieldwork (approval code FSREC 02 – 2016). Following an introduction to the research, informed consent was obtained from each respondent. Local permission was obtained from customary authorities on entering each village prior to conducting data collection. Respondent anonymity was preserved by recording responses based on respondent group identifiers and a unique number. For example, the second community member I interviewed from the village of Ifaty (IF) in the Bay of Ranobe (RA) was recorded as RAIF02. Partner organization (PO) respondents were recorded based on the country. For example, the third partner respondent interviewed in Guinea‐Bissau (GB) was recorded as GBPO3.

## RESULTS

### Identified bridging leaders

**FIGURE 4:**
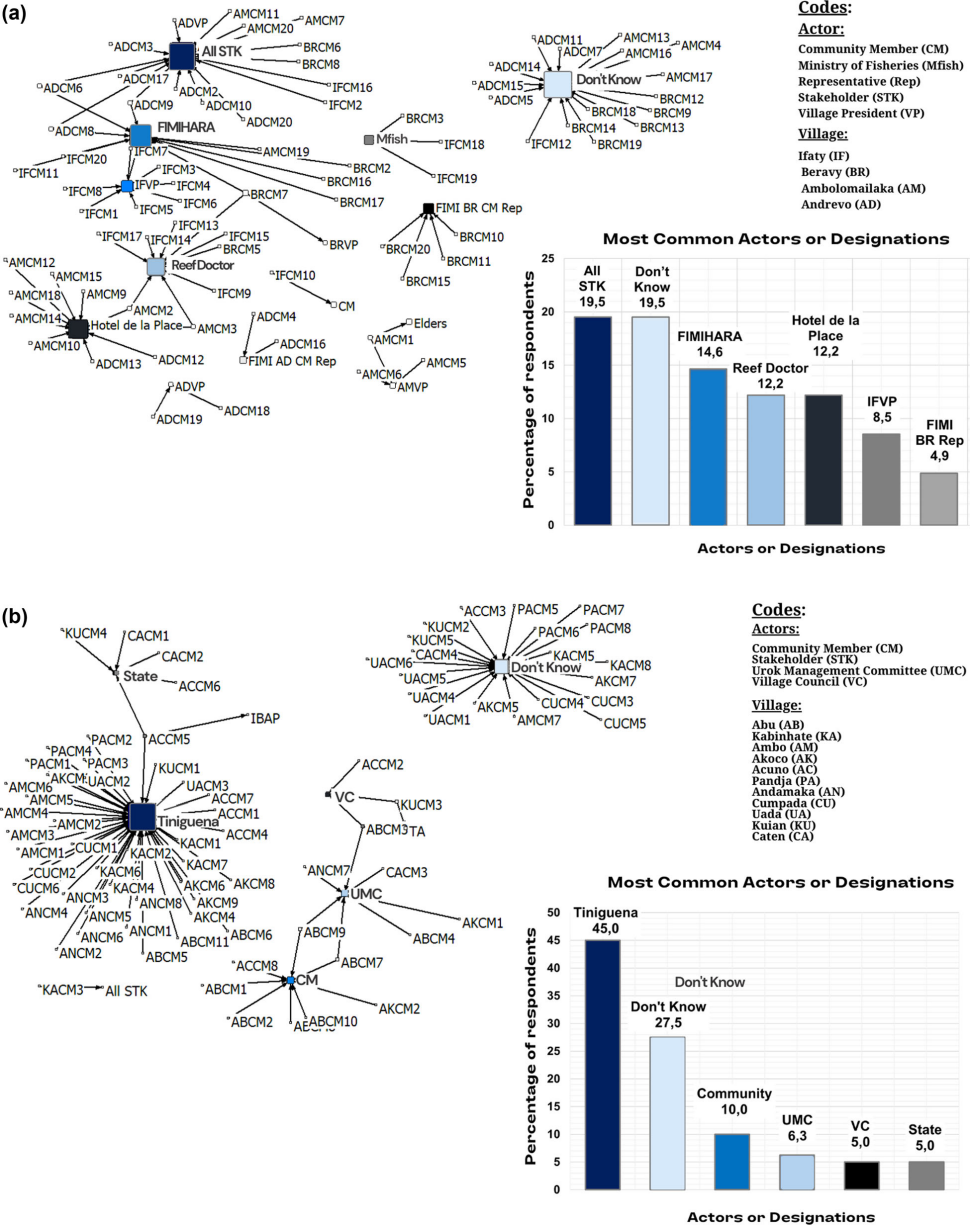
Social networks of actors community members identified as possessing the ultimate decision‐making power in (a) Bay of Ranobe and (b) the Urok Islands (the bigger the square icon, the greater the degree of centrality [i.e., more responses]). The total percentage of responses for key actors is also depicted.

Approximately, 19.5% of Bay of Ranobe and 27.5% of Urok respondents stated that they did not know who possessed the ultimate decision‐making power in their intervention (i.e., recorded as the actor designation don't know [Figure 4]). In the Urok Islands, this was most notable among villages located farther from the UMC headquarters in the village of Abu (e.g., approximately 67% of both Cumpada and Uada responses). Furthermore, 19.5% of Bay of Ranobe respondents selected the designation of all stakeholders as the ultimate decision makers (Figure 4a). However, both semistructured and informal group interviews confirmed that this was not the present case, but instead reflected the prevailing desire for greater communitywide involvement in future ICCA management.

The respective local conservation associations were identified as the ultimate decision makers by 14.6% of Bay of Ranobe respondents (i.e., FIMIHARA) (43% of Ifaty respondents) and 6.3% of Urok respondents (i.e., UMC) (33.3% of Akoco respondents) (Figure 4). Both partnering NGOs were frequently identified as ultimate decision makers (i.e., Reef Doctor and Tiniguena) (Figure 4), which was most strongly indicated among Urok respondents (i.e., approximately 45% identifying as Tiniguena) (Figure 4b). Responses that Tiniguena was the ultimate decision maker were most common in villages located close to their and the UMC's offices in Abu (e.g., approximately 57% of Ambo, 63% of Acuno, and 75% of Andamaka respondents) (Figure 4b). However, only about 27% of Abu respondents themselves identified Tiniguena as the ultimate decision maker; instead, they most frequently identified the community as a whole (i.e., 75.7% of respondents) (Figure 4b). Respondents from the Bay of Ranobe did not identify Reef Doctor to the same extent (i.e., 12.2% of responses), but did depict a similar village‐based pattern; a combined 77.7% of these responses came from the two villages located closest to Reef Doctor (i.e., Ifaty and Beravy) (Figure 4a).

Local village representatives were also frequently identified as ultimate decision makers (Figure 4). For example, for respondents from the Bay of Ranobe, approximately 35% of Ifaty responses identified their village presidents (Figure 4a). One Ifaty respondent stated, “If [the village president] says something needs to be done then the community will follow” (RAIF5). Similarly, representatives of the village councils emerged as key bridging leaders among the Urok Island respondents, most frequently in villages located farther west of Abu (Figure 4). One Cumpada respondent stated, “We respect them like our own father” (UICU4). Finally, both semistructured and informal group interviews revealed that village elders still possess recognition and status in the Urok Islands. A Kuian respondent noted that “If elders don't agree, it won't happen” (UIKU5).

### Appraisal of the identified bridging leaders

All bridging leaders appeared to be well positioned, particularly the local village representatives, and partner and local representatives appeared sufficiently knowledgeable to fulfill their bridging role. However, respondents in both cases revealed that sustained levels of self‐motivation and active engagement by leaders varied greatly, which they consistently attributed to a leader's perceptions of a lack of progress and benefits accruing from the interventions.

Identified partner and local leaders developed support networks with each other, and data obtained on community‐perceived relations indicated the development of local support needs improvement (Appendix S5). For example, many of the Urok respondents from villages located farther from Abu described their relations with the UMC as “poor” (e.g., 80% of Kuian respondents). In contrast, this group of Urok respondents perceived relations with their local village representatives as “good” (e.g., 100% of Cumpada, Kuian, and Caten respondents). However, perceived relations with both partner NGOs overall were positively perceived in the two communities, particularly in Tiniguena. That said, once again, a village‐based pattern did emerge with less positively perceived partner relations in the Urok villages located farther from Abu. A similar village‐based pattern of perceived relations with Reef Doctor emerged among Bay of Ranobe respondents from villages located farther from Ifaty.

It could be inferred from respondents in both cases that the aforementioned negative village‐based perceptions were largely due to a lack of initial involvement and perceived benefit‐sharing associated with their respective interventions. Elite capture was frequently emphasized by respondents; by implication, the level of integrity of many identified leaders was brought into question. For example, all partner respondents in Madagascar identified elite capture as a core constraining factor for CBC interventions in the country. Furthermore, Bay of Ranobe respondents commonly expressed a lack of confidence in FIMIHARA to manage finances equitably. This was most frequently cited in the northern Bay of Ranobe villages of Ambolomailaka and Andrevo, where approximately 70% and 80% of respondents, respectively, expressed a lack of trust in FIMIHARA. Similarly, in the Urok Islands, non‐Abu respondents frequently perceived elite capture by the village of Abu.

An additional exclusion‐related issue that community respondents identified was the perceived marginalization of minority cultural groups in both communities. This emerged most notably in the Bay of Ranobe between the predominantly Vezo population (i.e., traditionally fishers) and the Masikoro (i.e., traditionally farmers). Accordingly, Masikoro respondents frequently perceived their exclusion from representation on FIMIHARA, while many Vezo respondents expressed concern that the Masikoro lack necessary local ecological knowledge and often make use of destructive fishing methods. Consequently, these perceptions of the village‐ and culture‐based exclusion suggest that improved cultural awareness and empathy is required for leaders to more effectively motivate and empower others, establish a shared vision, manage conflicts, and facilitate inclusive engagement.

Respondents also emphasized that negative community perceptions were affecting the perceived legitimacy of their leaders and their ability to build trust and subsequently motivate others, establish a shared vision, communicate effectively, provide social support, manage conflict, and facilitate inclusive engagement. In both cases, respondents frequently attributed these negative perceptions to a lack of perceived initial involvement and, in particular, to ineffective communication, most notably a lack of knowledge diffusion by the identified local bridging leaders. Once again, this finding was most prominent for villages located farther from the respective intervention's headquarters. Accordingly, findings from the SRNA and interviews indicated knowledge exchange takes place predominantly between community members and within their own families. Given that community respondents in both cases stated that their local representatives were their only points of contact with partner organizations, the levels of effective communication between the identified bridging leaders and others require urgent improvement. Furthermore, this has implications for the effectiveness of the leadership characteristics of transparency and their leader's ability to support and empower others. That said, a lack of attendance at community meetings was observed in both cases, which is the primary forum for local representatives to exchange knowledge obtained from partner organizations with their constituents.

It could be inferred from community semistructured and informal group interviews that the perceived legitimacy (and trust) of identified leaders also has implications for their ability to act as role models relative to normatively desirable behavior and to provide social support. When asked in the SRNA to identify sources of social support, approximately 16% and 14.8% of community responses in the Bay of Ranobe identified Reef Doctor and FIMIHARA, respectively. At the village level, approximately 65% of Ifaty and 63% of Beravy respondents specifically identified their respective village presidents as their key source of social support. However, and perhaps more importantly, about 22.2% of all community members in the Bay of Ranobe stated that they did not trust any leader enough to approach the person for social support. Of the Urok respondents, approximately 20% identified the UMC, 25% identified both Tiniguena and their respective village council members, and 26.3% identified other community members as key sources of social support. Therefore, it can be inferred from these results that the ability of many of the identified bridging leaders to provide social support requires improvement.

## DISCUSSION

### Case study findings

Inclusive and equitable CBC interventions require multiple sources of leadership as the qualities of certain leaders may be polarizing (e.g., Alexander et al., 2018; Steenbergen & Warren, 2018). Both cases, and the CBC literature, emphasized partner organizations as important sources of bridging leadership (e.g., Berdej & Armitage, 2016). That said, both global CBC coastal‐marine research (e.g., Lyons & Cavaye, 2016; Steenbergen & Warren, 2018), and notable to the present discussion, their African equivalents (e.g., Mbaru & Barnes, 2017; Barnes et al., 2019), indicate how local leaders are often well positioned, and if their capacity is strengthened, they provide an ideal component for conservation success. Accordingly, while the main partners were frequently identified and positively perceived as important bridging leaders in both cases, several sources of local bridging leadership emerged, notably, local village presidents and councils.

The case studies revealed shortcomings in several of the leadership characteristics proposed in the framework (Figure 2). Respondents identified the ability to facilitate inclusive engagement as the central role of all bridging leaders. However, perceived ineffective communication emerged as inhibiting this process with implications for numerous other leadership characteristics in both cases, particularly, the ability to develop support networks. Although support networks are well‐developed between local representatives and partners, those between local leaders and their community members are less so. More specifically, a trend of community‐perceived village‐based exclusion associated with villages located further from the intervention's headquarters was revealed in both cases. Furthermore, perceived culture‐based exclusions emerged; particularly in the Bay of Ranobe between the predominantly Vezo population and other coastal‐migrant groups, such as the Masikoro. Even though Vezo identity is considered “fluid” and a “learned lifestyle” (Astuti, 1995), responses, and past studies, reveal that perceptions of pure versus not pure Vezo may cause conflict (Cripps & Gardner, 2016). Consequently, this reinforces the importance of cultural awareness for effective conservation leadership (Straka et al., 2018; Rice et al., 2021).

A lack of effective communication and inclusive engagement in both cases also appears to have implications for a leader's ability to build trust, be transparent and accountable, establish a shared vision, motivate and empower others, and manage conflicts. In particular, the findings concurred with past studies by emphasizing the importance of effective downward communication (i.e., from leaders to other community members) for more inclusive engagement, and conservation success (e.g., Barnes et al., 2019; Rice et al., 2022).

As introduced previously, the reviewed literature emphasized emotional intelligence as an important aspect of an effective leader (Alvesson & Einola, 2019). Although I acknowledge that the findings only begin to showcase the need for emotionally intelligent conservation leaders, both case studies did emphasize the importance of integrity and empathy.

Elite capture (i.e., the integrity of leaders) is a frequently cited, but not inevitable, constraint in CBC interventions (Warren & Visser, 2016; Rice et al., 2021). Nevertheless, the perceived elite capture in both cases requires minimizing through a greater understanding of the local power dynamics. Furthermore, the presence of multiple sources of effective bridging leadership may hold leaders more accountable to each other, and their constituents. Moreover, while context‐specific, the two cases indicate that the appointment of local bridging leaders, where possible, should be aligned with functioning and locally respected institutions (Golebie et al., 2022; Rice et al., 2021).

Findings also indicated bridging leaders require greater empathy to better consider and address the needs of minority groups, whether based on gender, culture, or village location. In addition, important leadership competencies, skills, and behaviors related to emotional intelligence alluded to in both cases included the need for greater transparency, provision of social support, and empowering of others. Accordingly, shortcomings in these characteristics could be improved through increased effective downward communication by leaders.

Two characteristics associated with emotionally intelligent leaders that did not emerge directly in the two cases were humility and optimism. The leadership literature emphasizes the centrality of humility to a leader's ability to engage, motivate, and empower others (e.g., Sousa & van Dierendonck, 2017), which is crucial to the collaborative governance contexts of CBC interventions. Accordingly, as introduced previously, Black (2021: 321–322) recently emphasized that conservation leaders should place “purpose before ego.” Therefore, I recommend further research into the humility of CBC leaders and their ability to produce intervention outcomes.

As McAfee et al. (2019: 280) state, “Optimism helps find the common ground for collaboration, uniting divergent groups with the hope that our collective efforts will achieve beneficial environmental outcomes.” Accordingly, optimism is particularly relevant to CBC contexts, and research shows that a self‐confident, self‐motivated, and actively engaged leader is better able to inspire greater levels of collective optimism for both their leadership and the intervention (e.g., Golebie et al., 2022; Rice et al., 2021). Therefore, this is another area of conservation leadership requiring further research.

As established throughout, emotional intelligence is a well‐recognized topic of importance in the leadership and management literature; however, scholars are divided on how it should be viewed and measured (Dasborough et al., 2021; Dong et al., 2022). Emotional intelligence research is problematic as it overlaps substantially with traditional personality factors, is often focused on the individual, and is complex, multidimensional, contextually grounded, and subject to bidirectional effects (Dong et al., 2022). Although focused on the individual leader, the present study takes a preliminary step toward showcasing the complex, multidimensional nature of emotional intelligence by unpacking it into several leadership characteristics. However, as emotional intelligence in conservation leadership remains a knowledge gap, I strongly encourage a future research agenda that builds on the foundation laid by other fields (e.g., Dasborough et al., 2021; Dong et al., 2022), to better understand and measure different factors and cross‐level effects of emotional intelligence on conservation outcomes.

The aforementioned research agenda should also consider several common qualitative research limitations experienced in the present study. First, my positionality could have affected data interpretation. Second, the limited time spent in the two case studies, and the methods I employed may have affected both my ability to sufficiently gain the trust of local respondents, and the comprehensiveness of data collected. Third, respondents may have been reluctant to express negative perceptions of their bridging leaders. For example, as Urok respondents frequently emphasized their dependency on partners for necessary development projects, they may have been hesitant to express negative perceptions toward them and their performance. Furthermore, responses regarding local leaders may have been affected by perceived local repercussions. Moreover, cultural norms could have influenced responses. For example, the Vezo respondents in the Bay of Ranobe may have been reluctant to express negative opinions due to their culturally nonconfrontational nature. Therefore, future research should be cognizant of these (and other) limitations. Notwithstanding these limitations, and while I do not advocate for the use of the social over natural sciences, nor qualitative over quantitative approaches, I join others in emphasizing that the social sciences have much to offer conservation management (e.g., Moon et al., 2019). That said, I believe future research into conservation leadership will benefit from an interdisciplinary approach that incorporates diverse approaches and a plurality of methods, and a robust research design process.

### Concluding remarks

Bridging leadership is a relational challenge *and* opportunity. Accordingly, an effective CBC bridging leader should understand and develop relational capital within the system to be lead. This requires understanding the institutional network structures, cultures, histories, and stakeholder perceptions in an intervention to minimize mistrust and misunderstandings, and manage conflict (Golebie et al., 2022; Rice et al., 2021). More specifically, this necessitates leaders able to *effectively communicate*, *provide social support*, and *empower others*, which, in turn, requires *self‐confident*, *self‐motivated*, and *actively engaged* leaders who possess *integrity*, *humility*, *optimism*, and *empathy* (Golebie et al., 2022; Rice et al., 2021). Furthermore, effective leadership also requires constant evaluation and capacity‐building. Moreover, since leaders are only as effective as their ability to positively influence others, evaluations should be inclusive. In addition, effective leadership requires leaders able to critically self‐evaluate, and constantly develop their leadership competency (e.g., Jansson et al., 2021).

To conclude, effective leadership is central to facilitating “unprecedented collaboration” required by the Post‐2020 framework. Although no best or perfect leader exists, the present discussion contributes toward a more in‐depth understanding of leadership complexities required for CBC, and other conservation interventions. Therefore, the discussion should be useful to diverse conservation actors. That said, this paper merely represents a starting point, and in particular, I strongly encourage a future research agenda focused on what constitutes an emotionally intelligent conservation bridging leader, and how this may influence the ability to deliver desirable post‐2020 ecological *and* social outcomes.

## Supporting information

Appendix S1: A summary of key leadership approaches, studies, and characteristics deemed relevant to community‐based conservation bridging leadership.

Appendix S2: Common conservation leadership characteristics.

Appendix S3: A purposive review of common effective leadership characteristics in community‐based conservation literature

Appendix S4: Social Relations and Network Appraisal Questionnaire, and Interview Guides

Appendix S5: Community‐perceived relations with identified bridging leaders
