# Supplementary material for: Identifying and developing effective post‐2020 conservation bridging leaders
Source: Conserv Biol. 2022 Oct 6;36(6):e13980. doi: 10.1111/cobi.13980 (PMC10092307; doi:10.1111/cobi.13980)
Supplement: Supplementary file 1 — Appendix S1: A summary of key leadership approaches, studies, and characteristics deemed relevant to community‐based conservation bridging leadership. [file COBI-36-0-s005.pdf]

Appendix S1: A summary of key leadership approaches, studies, and characteristics deemed relevant to community-based conservation bridging leadership.

Table: Key leadership approaches/ theories, studies, and characteristics deemed relevant to community-based conservation bridging leadership.

<table>
<tr><th rowspan="2">Approach</th><th rowspan="2">Definition</th><th colspan="3">Key leadership studies and characteristics relevant to
CBC bridging leadership</th></tr>
<tr></tr>
<tr><td rowspan="2">Trait Approach</td><td rowspan="2">Leadership traits can be defined "as relatively coherent and integrated patterns of personal characteristics, reflecting a range of individual differences, that foster consistent leadership effectiveness across a variety of group and organisational situations
(Zaccaro et al., 2004: p104).</td><td>Stogdill (1974)</td><td>Kirkpatrick & Locke (1991)</td><td>Zaccaro et al. (2004)</td></tr>
<tr><td>● achievement
● persistence
● insight
● initiative
● self-confidence
● responsibility
● cooperativeness
● tolerance
● influence
● sociability</td><td>● drive
● motivation
● integrity
● confidence
● cognitive ability
● task knowledge</td><td>● cognitive abilities
● extraversion
● conscientiousness
● emotional stability
● openness
● agreeableness
● motivation
● social intelligence
● self-monitoring
● emotional intelligence
● problem-solving</td></tr>
<tr><td rowspan="2">Skills Approach</td><td rowspan="2">The Skills Approach "is based on an individual's capability for solving the kind of novel, ill-defined problems with which people are presented in organisational leadership roles."
(Mumford et al., 2000: p156).</td><td>Katz (1955)</td><td>Mumford et al. (2000)</td><td></td></tr>
<tr><td>● technical skill in activity or work
● human skill to work with people
● conceptual skill to work with ideas to create a vision and strategy</td><td>● problem-solving skills
● social judgement skills
● perspective taking
● social perceptiveness
● behavioral flexibility
● social performance
● knowledge</td><td></td></tr>
</table>

Values-Based Leadership Theories				
**Definition:** “the role, task, and responsibility of values-based leaders is to help followers realise the most important ends that they hold dear but cannot obtain by themselves.” (O’Toole, 2008: p7).				
Servant Leadership	Servant leaders “combine their motivation to lead with a need to serve.” (Van Dierendonck, 2011: p1228).	**Laub (1999)**	**Wong & Davey(2007)**	**Van Dierendonck (2011)**
		● Developing people<br>● Sharing Leadership<br>● Displaying authenticity<br>● Valuing people<br>● Providing leadership<br>● Building community	● Serving and developing others<br>● Consulting and involving others<br>● Humility and selflessness<br>● Modelling integrity and authenticity<br>● Inspiring and influencing others	● Empowering and developing people<br>● Humility<br>● Authenticity<br>● Interpersonal Acceptance<br>● Providing Direction<br>● Self-Determination<br>● Moral Cognitive Development<br>● Need to serve and Motivation to Lead<br>● Stewardship
Authentic Leadership	Authentic leaders are “those who are deeply aware of how they think and behave and are perceived by others as being aware of their own and others’ values/moral perspectives, knowledge, and strengths; aware of the context in which they operate; and who are confident, hopeful, optimistic, resilient, and of high moral character” (Avolio et al., 2004a: p4).<br>Avolio et al. (2004b: p806) asserts that authentic leaders “act in accordance with deep personal values and convictions, to build credibility and win the respect and trust of followers”.	**George (2003)**	**Luthans & Avolio (2003)**	**Avolio & Gardner (2005)**
		● Purpose<br>● Values<br>● Relationships<br>● Self-Discipline<br>● Heart	● Confident<br>● Hopeful<br>● Optimistic<br>● Resilient<br>● Transparent<br>● Moral/ Ethical<br>● Future-Oriented<br>● Associate Building	● **Leader self-awareness**<br>▪ *Values*<br>▪ *Cognitions*<br>▪ *Emotions*<br>● **Leader self-regulation**<br>▪ *Internalized*<br>▪ *Balanced processing*<br>▪ *Relational transparency*<br>▪ *Authentic behavior*<br>● **Leadership processes/behaviors**<br>▪ *Positive modelling*<br>▪ *Personal and social identification*<br>▪ *Emotional contagion*<br>▪ *Supporting self-determination*<br>▪ *Positive social exchanges*

Ethical Leadership	Ethical leadership is “the demonstration of normatively appropriate conduct through personal actions and interpersonal relationships, and the promotion of such conduct to followers through two-way communication, reinforcement, and decision-making” (Brown et al., 2005: p120).	**Brown & Treviño (2006)**	**Northouse (2016)**	
		● Altruism<br>● Ethical decision-making<br>● Integrity<br>● Role modelling<br>● Moral management	● Respects others<br>● Serves others<br>● Shows justice<br>● Manifest honesty<br>● Builds community	
Spiritual Leadership	“The values, attitudes, and behaviors that are necessary to intrinsically motivate one’s self and others so that they have a sense of spiritual survival through calling and membership” (Fry, 2003: p711).	**Fry (2003)**	**Fry & Whittington (2005)**	**Reeve (2005)**
		● **Vision**<br>▪ *Broad appeal to key stakeholders*<br>▪ *Defines the destination and journey*<br>▪ *Reflects high ideals*<br>▪ *Encourage hope/ faith*<br>▪ *Establishes a standard of excellence*<br>● **Altruistic love**<br>▪ *Trust/ loyalty*<br>▪ *Forgiveness/ Acceptance/ Gratitude*<br>▪ *Integrity*<br>▪ *Honesty*<br>▪ *Courage*<br>▪ *Humility*<br>▪ *Kindness*<br>▪ *Empathy/ compassion*<br>▪ *Patience/ Meekness/ Endurance*<br>● **Hope/ faith**<br>▪ *Endurance*<br>▪ *Perseverance*<br>▪ *Do what it takes*<br>▪ *Stretch goals*<br>▪ *Expectation of reward/ victory*	<u>**Legacy Leadership Model**</u><br>● **Leadership Motives/ Methods/ Measures**<br>▪ *Pure Motives*<br>▪ *Authentic/ Sincere*<br>▪ *Follower-Centered; Not Self-Centered*<br>▪ *Affectionate/ Emotional*<br>▪ *Worthy of Imitation*<br>▪ *Boldness Amid Opposition*<br>▪ *Influence Without Asserting Authority*<br>▪ *Active; not Passive*<br>▪ *Vulnerable/ Transparent*	● Integrity<br>● Honesty<br>● Humility<br>● Respect for others<br>● Fair treatment<br>● Caring and concern<br>● Listening<br>● Appreciating others<br>● Reflective practice

Literature cited in table:

1. Avolio, B.J., & Gardner, W.L. (2005). Authentic leadership development: Getting to the root of positive forms of leadership. *The Leadership Quarterly*, 16(3), 315-338. https://doi.org/10.1016/j.leaqua.2005.03.001
2. Avolio, B., Luthans, F., & Walumbwa, F.O. (2004a). *Authentic leadership: Theory-building for veritable sustained performance.* Working paper. Gallup Leadership Institute, University of Nebraska, Lincoln.
3. Avolio, B.J., Gardner, W.L., Walumbwa, F.O., Luthans, F., & May, D.R. (2004b). Unlocking the mask: A look at the process by which authentic leaders impact follower attitudes and behaviors. *The Leadership Quarterly*, *15*, 801–823. https://doi.org/10.1016/j.leaqua.2004.09.003
4. Brown, M.E., Treviño, L.K., & Harrison, D. (2005). Ethical leadership: A social learning perspective for construct development and testing. *Organizational Behavior and Human Decision Processes*, 97, 117−134. https://doi.org/10.1016/j.obhdp.2005.03.002
5. Fry, L.W. (2003). Toward a theory of spiritual leadership. *The Leadership Quarterly*, 14(6), 693-727. https://doi.org/10.1016/j.leaqua.2003.09.001
6. Fry, L.W., & Whittington, J.L. (2005). *Spiritual leadership as a paradigm for organization transformation and development.* 65th Annual Meeting of the Academy of Management, Honolulu, Hawaii, pp. 5-10. https://iispiritualleadership.com/wp-content/uploads/docs/SLTAOM2005OrgDev.pdf
7. George, B. (2003). *Authentic leadership: Rediscovering the secrets to creating lasting value*. San Francisco: John Wiley & Sons.
8. Katz, R. (1955). Skills of an effective administrator. *Harvard Business Review*, 33(1), 33-42.
9. Kirkpatrick, S.A., & Locke, E.A. (1991). Leadership: do traits matter?. *Academy of management perspectives*, 5(2), 48-60.
10. Luthans, F., & Avolio, B. J. (2003). *Authentic leadership development*. In: Kim, S., Cameron, Dutton, J.E., & Quinn, R.E. (Eds.). *Positive organizational scholarship: Foundations of a New Discipline*. San Francisco: Berrett-Koehler, pp. 241-258.
11. Mumford, M.D., Zaccaro, S.J., Connelly, M.S., & Marks, M.A. (2000). Leadership skills: Conclusions and future directions. *The Leadership Quarterly*, 11(1), 155-170. https://doi.org/10.1016/S1048-9843(99)00047-8
12. Northouse, P. (2016). *Leadership Theory and Practice.* (7th edition). Thousand Oaks: Sage.

13. O'Toole, J. (2008). Notes Toward a Definition of Values-Based Leadership. *The Journal of Values-Based Leadership*, 1(1), Article 10. http://scholar.valpo.edu/jvbl/vol1/iss1/1
14. Pearce, C.L., & Conger, J.A. (2003). *All those years ago: The historical underpinnings of shared leadership.* In: Pearce, C.L., & Conger, J.A. (Eds.). *Shared Leadership: Reframing the Hows and Whys of Leadership*. Thousand Oaks: Sage, pp. 1–18.
15. Reave, L. (2005). Spiritual values and practices related to leadership effectiveness. *The Leadership Quarterly*, 16(5), 655-687. https://doi.org/10.1016/j.leaqua.2005.07.003
16. Stogdill, R.M. (1974). *Handbook of leadership: A survey of theory and research*. Free Press.
17. Van Dierendonck, D. (2011). Servant leadership: A review and synthesis. *Journal of management*, 37(4), 1228-1261. https://doi.org/10.1177%2F0149206310380462
18. Wong, P.T., & Davey, D. (2007). *Best practices in servant leadership.* Servant Leadership Research Roundtable, School of Global Leadership and Entrepreneurship, Regent University, July 2007. http://www.regent.edu/acad/global/publications/sl_proceedings/2007/wong-davey.pdf
19. Zaccaro, S.J., Kemp, C., & Bader, P. (2004). *Leader traits and attributes.* In: Antonakis, J.E., Cianciolo, A.T., & Sternberg, R.J. (Eds.), *The nature of leadership.* Thousand Oaks, CA: Sage Publications, pp. 101-124.
20. Zhu, J., Liao, Z., Yam, K.C., & Johnson, R.E. (2018). Shared leadership: A state‐of‐the‐art review and future research agenda. *Journal of Organizational Behavior*, *39*(7), 834-852. https://doi.org/10.1002/job.2296
