# Supplementary material for: Identifying and developing effective post‐2020 conservation bridging leaders
Source: Conserv Biol. 2022 Oct 6;36(6):e13980. doi: 10.1111/cobi.13980 (PMC10092307; doi:10.1111/cobi.13980)
Supplement: Supplementary file 2 — Appendix S2: Common conservation leadership characteristics. [file COBI-36-0-s003.pdf]

Appendix S2: Common conservation leadership characteristics.

The figure below summarizes the common leadership characteristics identified within the conservation practitioner literature reviewed.

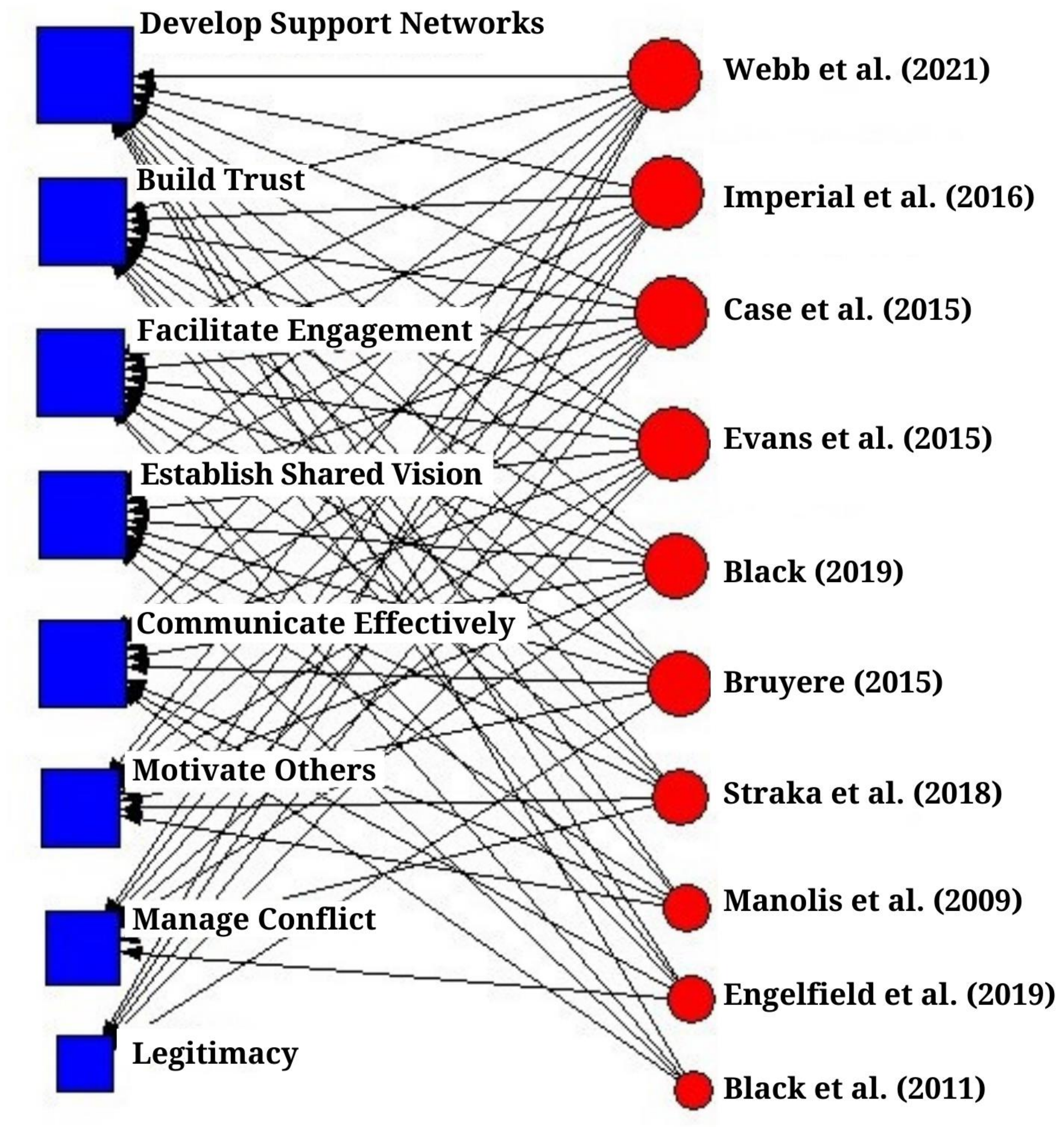

Figure: A social network map depiction of common characteristics of effective leadership emerging from conservation practitioner literature. Note: The square icons decrease in size to depict the decreasing frequency of the characteristics based upon 'degree of centrality' (i.e., the bigger the square the greater the number of times the characteristic is mentioned in the respective studies).

The specific findings as presented by the authors of each paper depicted in the figure above can be found below:

A. **Webb et al. (2021: p167-169)** identify a set of five leadership domains, each with leadership practices, that contribute to positive conservation outcomes based upon a systematic review:

**Leadership Domains**	**Leadership Practices**
1. Stakeholder Engagement	• Extending access to stakeholders to influence;<br>• Decision-making, sufficient communication with stakeholders;<br>• Addressing conflict effectively and establishing;<br>• Clear roles for partners
2. Trust	• Investing time to become familiar with local context and culture;<br>• Taking time to build relationships and understand stakeholders' needs, values and concerns;<br>• Facilitating a two-way exchange of knowledge with the local community;<br>• Interpersonal and cross-cultural skills
3. Vision	• Collaboratively develop and articulate a vision that fosters innovation;<br>• Integrating the input of external groups/ stakeholders to build the vision
4. Individual champion	• Persisting through challenging periods;<br>• Demonstrating unwavering passion for the conservation cause;<br>• Inspiring others to act
5. Excellence in internal attributes	• Exhibiting clear and effective communication with staff or team members;<br>• Demonstrating the ability to understand and/or address the conservation issue at different scales;<br>• Adapting to changing circumstances

B. **Imperial et al. (2016: p128)** discuss three interconnected types of leadership in large landscape conservation contexts characterised by network settings (i.e., collaborative distributive; and architectural leadership). In particular, they emphasize the need to move away from traditional (bureaucratic) leadership to collaborative leadership.

Traditional leadership	*Collaborative leadership*
Vision is possessed and articulated by the leader	Helps craft collective vision
Leader frames the problem and solution for followers	Helps others frame a collective definition of the problem and appropriate solutions
Leader has to have followers to lead	Leader is simultaneously a follower
Unilateral decision making based on hierarchy, formal position, or legal authority	Shared decisions and values
Communication within a single organization or homogenous group with shared interests or values	Communication across diverse groups with competing interests and values
Working within boundaries (eg program, organization, jurisdiction)	Working across boundaries
Focus on certainty	Tolerates and embraces ambiguity and complexity
Leader directs action	Leader facilitates and coordinates shared action
More closely aligned with transactional theories of leadership	More closely aligned with charismatic or transformational theories of leadership

C. Both **Case et al. (2015)** and **Evans et al. (2015)** systematically review research on differing constructions and characterizations of leaders and leadership in environmental leadership. Case et al. (2015) emphasize the need for more critical research on environmental leadership that borrows from political ecology to better understand how leadership practices: (a) reflect culturally complex and plural contexts, and; (b) result in different types of outcome. Evans et al. (2015) conclude this body of research is critically analysing: 1) multiple, interacting leaders, (2) leadership practices and processes, (3) leadership in different contexts, and (4) leadership outcomes from different perspectives. Please consult these papers further if interested.

D. **Black (2019: p132)** reviews Black et al.'s (2011) aforementioned qualities and abilities of an effective conservation leader as it relates to established findings with the *New Psychology of Leadership* literature. He emphasizes that Black et al.'s (2011) findings remain a robust guide for wildlife conservation leadership, and summarizes the relevant assertions for conservation leaders as:

1) Provide clarity and agreement on purpose, vision and goals;
2) Define organisational boundaries and capacity to influence externally;
3) Be sensitive to people, their culture, expectations, strengths and identity;
4) Have confidence to engage dissent and people with alternative views;
5) Work alongside people in cooperation and partnership;
6) Give people more control through clear governance and flexible plans;
7) Inform decisions with data, and learn from failures without blame;
8) Recognise and celebrate success and encourage pursuit of improvements.

E. **Bruyere (2015: p379)** developed an importance rating of 20 potential skills for conservation leaders based upon interviews, a review of literature, and an online survey (i.e., N=103).

**Skill**	**Cumulative "agree" and "strongly agree" per cent**
Partnership-building	93
Establishing a vision	89
Conflict management	83
Situation assessment	83
Strategic planning	83
Facilitation skills	77
Creating a positive organizational culture	77
Solving problems	77
Community outreach	72
Motivating others	67
Development & fundraising	67
Conservation planning	58
Managing/supervising employees	58
Risk assessment	58
Mentoring	52
Systems thinking	50
Public relations	44
Scientific expertise	33
Research skills	11

F. **Straka et al. (2018: p114)** systematically review and confirm the conservation leadership attributes identified by Bruyere (2015), and in doing so add *effective communication.* In particular, they emphasize the importance of *motivating others*, *establishing a shared vision*, and *effective communication* (i.e., in 80, 67, and 60% of studies included). Furthermore, they emphasize the specific need to account for how cultural context influences conservation leaders. Moreover, they propose that conservation leadership training needs to focus on the following aspects:

1) Exploring the explicit relationship between conservation leadership attributes and cultural context, as this can help to understand the effectiveness of conservation actions.
2) Identifying the drivers of values, motivation, communication styles and group dynamics when working in a particular country, region or with a specific group of people.
3) Providing conservation leadership training and education that includes cultural awareness and opportunities to learn in situ.

G. **Manolis et al. (2009: p881)** summarize a set of 8 adaptive-leadership principles (with some modification of terminology) that we believe applies to conservation-science leadership, recognizing that no single model or set of concepts is universally applicable: recognize the social dimension of the problem; cycle frequently through action and reflection; get and maintain attention; combine strengths of multiple leaders; extend influence through networks of relationships; time efforts strategically; nurture productive conflict; and cultivate diversity

H. **Englefield et al. (2019)** develop a list of 15 positive and 3 negative competencies and qualities of effective leaders reported as important by conservation leaders in an online questionnaire (i.e., N=130).

**Leadership competencies**	**Mean ($\bar{x}$)**	**Standard deviation (σ)**
1. Rally their team based on trust, not fear	4.75	0.45
2. Create an inspirational vision	4.69	0.49
3. Demonstrate the behaviours they expect to see in others	4.60	0.58
4. Enable others around them to act, succeed and grow	4.60	0.95
5. Ensure effective communication within the team	4.59	0.96
6. Connect with their followers by recognising contributions and celebrating successes	4.49	0.56
7. Encourage growth and innovation	4.42	0.83
8. Be reliable and consistent in decision making	4.31	0.86
9. Be aware of benefits of adapting to the needs of their team	4.24	0.83
10. Be self-aware of how they are as a leader	4.19	1.01
11. Be a mentor for others	4.15	1.03
12. Provide team members the opportunity to pursue the actions they think will be effective	4.12	0.8
13. Look to a mentor when support or guidance is needed	4.03	1.01
14. Have conservation as a primary passion	3.91	1.01
15. Demonstrate considerable experience within the sector	3.53	0.96
16. *Be as focused on achieving their own personal goals as the goals of the project, organisation or greater conservation action*	2.60	1.31
17. *Be the sole decision maker*	1.90	0.99
18. *Maintain a level of distance and detachment from the team*	1.75	0.91

I. **Black et al. (2011: p337)** synthesize a comprehensive list of the qualities and abilities of an effective conservation leader.

**Vision and goals**
• Establish a stable, shared long-term vision and a common sense of purpose.<br>• Identify what is happening to, or affecting, biodiversity (populations, productivity, threats).<br>• Set clear, short-term achievable goals.<br>• Ensure flexibility in all levels of planning.<br>• Consider view of stakeholders and partners.<br>• Ensure planning starts with understanding current performance relative to program purpose.<br>• Ensure that staff embrace project aims and culture (vision, understanding the system, goals).<br>• Get people to measure performance in relation to project aims.<br>• Advocate good governance, particularly in large complex projects.<br>• Ensure congruency between plans, action on the ground and results.
**Hands-on leadership**
• Be orientated toward "hands-on" management, working with staff.<br>• Possess highly developed biological and/or operational skills appropriate to the program<br>• Be able to prioritize the work by asking key questions.<br>• Know people's strengths; channel their energy and passion to maximum effect.<br>• Understand cultural differences and manage people's expectations and viewpoints sensitively.<br>• Check results with staff and empower them to get the job done.<br>• Involve the people doing the work in data analysis, decisions, and implementing changes.<br>• Place responsibility and control of information in the hands of people who do the work.<br>• Ensure that an understanding of what matters to biodiversity steers the work people do.<br>• Have two-way communication meetings, with an emphasis on clarifying, testing, and listening.<br>• Ensure managers lead; spend time with staff, listen to concerns, and enable contributions.
**Consider both project details and the big picture**
• Focus both internally and externally, understanding intra- and inter-organizational dynamics.<br>• Know projects' sphere of influence—identify the solvable problems.<br>• Establish budgets and a clear fund-raising strategy.<br>• Examine financial and nonfinancial measures; which predict and cause conservation results?<br>• Base information, technology, and resource needs on how they help people's core work.<br>• Create an attitude of cooperation with project partners, sharing information to improve work.<br>• Anticipate unexpected outcomes.<br>• Be prepared to seek specialist advice from external sources.<br>• Integrate management flexibility alongside professional/scientific rigor.<br>• Determine whether data on staff, communities, or society would be useful for the program.
**Improvement and Learning**
• Give people the opportunity to ask for training and provide it on a just-in-time basis.<br>• Be receptive to (and seek out) alternative solutions.<br>• Enable staff to challenge, share, and learn from mistakes, without fear.<br>• Expect—and support staff to strive for—high standards.<br>• Expect the project (and its needs) to evolve through time.<br>• Understand risk factors and make suitable contingencies.<br>• Appraise the system rather than people; manage morale, celebrate success, learn from failures.<br>• Make improvements based on biodiversity needs and process performance, not arbitrary targets.<br>• Recognize difference between neglect and lack of capability (training, experience, or resources).<br>• Allow people doing the work, the freedom to experiment with methods to improve performance.

Literature Cited:

1. Black, S.A. (2019). Psychological knowledge relevant to leadership in wildlife conservation. *Open Journal of Leadership*, *8*(3), 114-141. https://doi.org/10.4236/ojl.2019.83007
2. Black, S.A., Groombridge, J.J., & Jones, C.G. (2011). Leadership and conservation effectiveness: finding a better way to lead. *Conservation Letters*, 4(5), 329-339. https://doi.org/10.1111/j.1755-263X.2011.00184.x
3. Bruyere, B.L. (2015). Giving Direction and Clarity to Conservation Leadership. *Conservation Letters*, 8(5), 378–3. https://doi.org/10.1111/conl.12174
4. Case, P., Evans, L.S., Fabinyi, M., Cohen, P.J., Hicks, C.C., Prideaux, M., & Mills, D.J. (2015). Rethinking environmental leadership: The social construction of leaders and leadership in discourses of ecological crisis, development, and conservation. *Leadership*, 11(4), 396-423. https://doi.org/10.1177%2F1742715015577887
5. Englefield, E., Black, S.A., Copsey, J.A., & Knight, A.T. (2019). Interpersonal competencies define effective conservation leadership. *Biological Conservation*, 235, 18-26. https://doi.org/10.1016/j.biocon.2019.03.043
6. Evans, L.S., Hicks, C.C., Cohen, P.J., Case, P., Prideaux, M., & Mills, D.J. (2015). Understanding leadership in the environmental sciences. *Ecology and Society*, 20(1). http://dx.doi.org/10.5751/ES-07268-200150
7. Imperial, M.T., Ospina, S., Johnston, E., O'Leary, R., Thomsen, J., Williams, P., & Johnson, S. (2016). Understanding leadership in a world of shared problems: advancing network governance in large landscape conservation. *Frontiers in Ecology and the Environment*, 14(3), 126-134. https://doi.org/10.1002/fee.1248
8. Manolis, J.C., Chan, K.M., Finkelstein, M.E., Stephens, S., Nelson, C.R., Grant, J.B., & Dombeck, M.P. (2009). Leadership: a new frontier in conservation science. *Conservation Biology*, 23(4), 879-886. https://doi.org/10.1111/j.1523-1739.2008.01150.x
9. Straka, T.M., Bal, P., Corrigan, C., Di Fonzo, M.M., & Butt, N. (2018). Conservation leadership must account for cultural differences. *Journal for Nature Conservation*, 43, 111-116. https://doi.org/10.1016/j.jnc.2018.03.003
10. Webb, S.A., Bruyere, B., Halladay, M., & Walker, S. (2021). A framework for conceptualizing leadership in conservation. *Oryx*, 1-7. https://doi.org/10.1017/S0030605320000629
