# Supplementary material for: Identifying and developing effective post‐2020 conservation bridging leaders
Source: Conserv Biol. 2022 Oct 6;36(6):e13980. doi: 10.1111/cobi.13980 (PMC10092307; doi:10.1111/cobi.13980)
Supplement: Supplementary file 3 — Appendix S3: A purposive review of common effective leadership characteristics in community‐based conservation literature [file COBI-36-0-s001.pdf]

Appendix S3: A purposive review of common effective leadership characteristics in community-based conservation literature

Search String:

The following search string was used in *Google Scholar* within the date range of *2000-2021*:

((("community-based" AND (conservation OR "natural resource management")) AND (leadership AND (traits OR values OR attributes OR qualities OR competencies OR skills OR behaviours)))

Inclusion criteria:

An included study described or suggested effective leadership characteristics – inclusive of attributes, skills, competencies, qualities and behaviours – within their context. Whilst many studies mentioned the need for 'strong leadership', these were not included. Upon inclusion of 54 studies described below, it was determined that theoretical saturation was met as no new characteristics were emerging.

Composition of included studies

Table: Composition of the 54 purposively reviewed community-based conservation studies by region and sector. Note: One study analyzed case studies in *Hawaii* and *American Samoa,* and was categorized as both an *Oceania* and *North America* example in the table.

**Region (*N*)**	**Sector**		
	Cross-Sector (N = 11)	*Terrestrial (N = 9)*	*Coastal-Marine (N = 34)*
**Global (10)**	7	0	3
**Africa (15)**	2	4	9
**Asia (13)**	2	2	9
**Caribbean (3)**	0	0	3
**Europe (2)**	0	1	1
**North America (2)**	0	0	2
**Oceania (7)**	0	1	6
**South America (3)**	0	0	3

Analysis:

Included studies were reviewed based on the eight leadership characteristics identified in the broader conservation literature: *develop support networks*, *build trust*, *facilitate engagement*, *establish a shared vision*, *communicate effectively*, *motivate others*, *manage conflicts*, and *legitimacy* (see *Supplementary Material S2* further). Thereafter, additional characteristics were identified and added to produce the thirteen included. The frequency of characteristics mentioned in the included studies was recorded in a two-dimensional matrix in *Excel,* and then developed into datasets, and subsequently, a social network map (i.e., Figure below) using *UCINET 6 Social Network Analysis* and *Netdraw* software, respectively (Borgatti et al., 2002[1]).

---

[1] Borgatti, S.P., Everett, M.G., & Freeman, L.C. (2002). *Ucinet 6 for Windows: Software for Social Network Analysis.* Harvard, MA: Analytic Technologies.

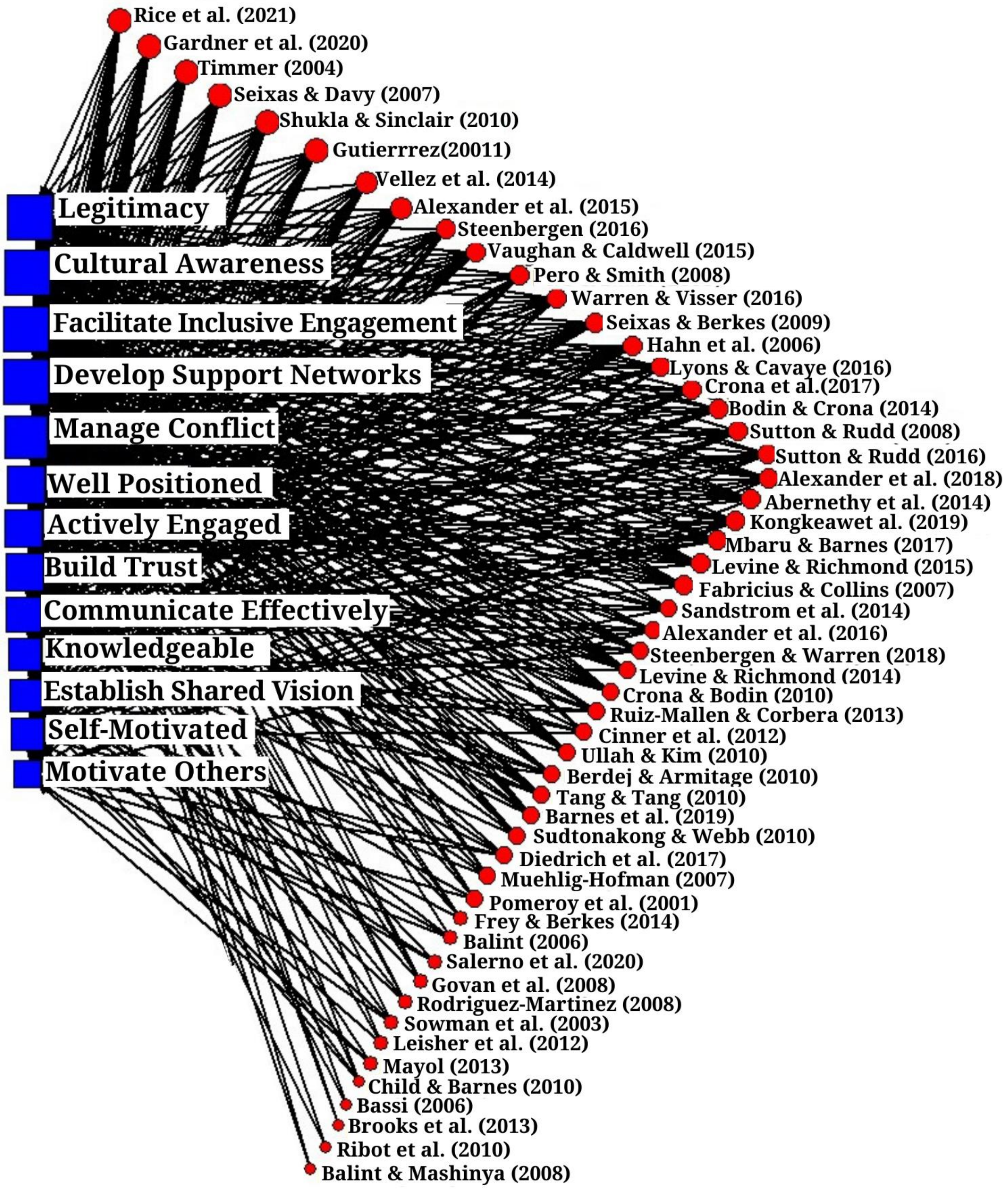

Figure: A social network map depiction of common effective leadership characteristics emerging from the 54 purposively reviewed community-based conservation studies. Note: The blue square icons decrease in size to depict the decreasing frequency of the identified characteristics based upon its 'degree of centrality' (i.e., the bigger the square the greater the number of times the characteristic is mentioned by the respective studies). Similarly, the red circles indicate the 54 included studies and decrease in size based on the number of characteristics mentioned by the respective study.

Case References:

1. Abernethy, K.E., Bodin, Ö., Olsson, P., Hilly, Z., & Schwarz, A. (2014). Two steps forward, two steps back: the role of innovation in transforming towards community-based marine resource management in Solomon Islands. *Global Environmental Change*, 28, 309-321. https://doi.org/10.1016/j.gloenvcha.2014.07.008

2. Alexander, S. M., Armitage, D., & Charles, A. (2015). Social networks and transitions to co-management in Jamaican marine reserves and small-scale fisheries. *Global Environmental Change*, 35, 213-225. https://doi.org/10.1016/j.gloenvcha.2015.09.001

3. Alexander, S. M., Andrachuk, M., & Armitage, D. (2016). Navigating governance networks for community-based conservation. *Frontiers in Ecology and the Environment*, *14*(3), 155-164. https://doi.org/10.1002/fee.1251

4. Alexander, S.M., Bodin, Ö., & Barnes, M. (2018). Untangling the drivers of community cohesion in small-scale fisheries. *International Journal of the Commons*, 12(1). http://doi.org/10.18352/ijc.843

5. Balint, P.J. (2006). Improving Community-Based Conservation Near Protected Areas: The Importance of Development Variables. *Environmental Management*, 38, 137–148. https://doi.org/10.1007/s00267-005-0100-y

6. Balint, P. J., & Mashinya, J. (2008). CAMPFIRE during Zimbabwe's national crisis: Local impacts and broader implications for community-based wildlife management. *Society and Natural Resources*, 21(9), 783-796. https://doi.org/10.1080/08941920701681961

7. Barnes, M.L., Mbaru, E., & Muthiga, N. (2019). Information access and knowledge exchange in co-managed coral reef fisheries. *Biological Conservation*, 238, 108198. DOI: https://doi.org/10.1016/j.biocon.2019.108198

8. Bassi, M. (2006). Community conserved areas in the Horn of Africa. *Parks*, 16(1), 28-34.

9. Berdej, S.M., & Armitage, D.R. (2016). Bridging organizations drive effective governance outcomes for conservation of Indonesia's marine systems. *PloS one*, 11(1), e0147142. https://doi.org/10.1371/journal.pone.0147142

10. Bodin, Ö., & Crona, B.I. (2008). Management of Natural Resources at the Community Level: Exploring the Role of Social Capital and Leadership in a Rural Fishing Community. *World Development*, 36(12), 2763-2779. https://doi.org/10.1016/j.worlddev.2007.12.002

11. Brooks, J., Waylen, K.A., & Mulder, M.B. (2013). Assessing community-based conservation projects: a systematic review and multilevel analysis of attitudinal, behavioral, ecological, and economic outcomes. *Environmental Evidence*, 2(1), 2. https://doi.org/10.1186/2047-2382-2-2

12. Child, B., & Barnes, G. (2010). The conceptual evolution and practice of community-based natural resource management in southern Africa: past, present and future. *Environmental Conservation*, 37(3), 283-295.

13. Cinner, J.E., Daw, T.M., McClanahan, T.R., Muthiga, N., Abunge, C., Hamed, S., ... & Jiddawi, N. (2012). Transitions toward co-management: the process of marine resource management devolution in three east African countries. *Global Environmental Change*, 22(3), 651-658. https://doi.org/10.1016/j.gloenvcha.2012.03.002

14. Crona, B., & Bodin, Ö. (2010). Power asymmetries in small-scale fisheries: a barrier to governance transformability?. *Ecology and Society*, 15(4). http://www.ecologyandsociety.org/vol15/iss4/art32/

15. Crona, B., Gelcich, S., & Bodin, Ö. (2017). The importance of interplay between leadership and social capital in shaping outcomes of rights-based fisheries governance. *World Development*, 91, 70–83. https://doi.org/10.1016/j.worlddev.2016.10.006
16. Diedrich, A., Stoeckl, N., Gurney, G.G., Esparon, M., & Pollnac, R. (2017). Social capital as a key determinant of perceived benefits of community-based marine protected areas. *Conservation Biology*, 31(2), 311-321. https://doi.org/10.1111/cobi.12808
17. Fabricius, C., & Collins, S. (2007). Community-based natural resource management: governing the commons. *Water Policy*, 9(S2), 83–97. https://doi.org/10.2166/wp.2007.132
18. Frey, J., & Berkes, F. (2014). Can partnerships and community-based conservation reverse the decline of coral reef social-ecological systems?. *International Journal of the Commons*, 8(1), 26–46. http://doi.org/10.18352/ijc.408
19. Gardner, C.J., Cripps, G., Day, LP., Dewar, K., Gough, C., Shawn Peabody, S., Tahindraza, G., & Harris, A. (2020). A decade and a half of learning from Madagascar's first locally managed marine area. *Conservation Science and Practice*, 2, e98: https://doi.org/10.1111/csp2.298
20. Govan, H., Aalbersberg, W., Tawake, A., & Parks, J. (2008). Locally managed marine areas: A guide to supporting community-based adaptive management. The Locally-Managed Marine Area (LMMA) Network. https://agris.fao.org/agris-search/search.do?recordID=GB2013203237
21. Gutiérrez, N.L., Hilborn, R., & Defeo, O. (2011). Leadership, social capital and incentives promote successful fisheries. *Nature, 470*(7334), 386-389. https://doi.org/10.1038/nature09689
22. Hahn, T., Olsson, P., Folke, C., & Johansson, K. (2006). Trust-building, knowledge generation and organizational innovations: the role of a bridging organization for adaptive comanagement of a wetland landscape around Kristianstad, Sweden. *Human ecology*, 34(4), 573-592. https://doi.org/10.1007/s10745-006-9035-z
23. Kongkeaw, C., Kittitornkool, J., Vandergeest, P., & Kittiwatanawong, K. (2019). Explaining success in community based mangrove management: Four coastal communities along the Andaman Sea, Thailand. *Ocean & Coastal Management*, 178, 104822. https://doi.org/10.1016/j.ocecoaman.2019.104822
24. Leisher, C., Hess, S., Boucher, T.M., van Beukering, P., & Sanjayan, M. (2012). Measuring the impacts of community-based grasslands management in Mongolia's Gobi. *PLoS One*, *7*(2), e30991. https://doi.org/10.1371/journal.pone.0030991
25. Levine, A.S., & Richmond, L.S. (2014). Examining enabling conditions for community-based fisheries comanagement: comparing efforts in Hawai‘i and American Samoa. *Ecology and Society*, 19(1), 24. http://dx.doi.org/10.5751/ES-06191-190124
26. Levine, A.S., & Richmond, L.S. (2015). Using common-pool resource design principles to assess the viability of community-based fisheries co-management systems in American Samoa and Hawai'i. *Marine Policy*, 62, 9-17. https://doi.org/10.1016/j.marpol.2015.08.019
27. Lyons, I., & Cavaye, J. (2016). Community-Led Engagement With Government and the Role of Community Brokers in East New Britain, Papua New Guinea. *Society & Natural Resources*, 29(4), 462-478. https://doi.org/10.1080/08941920.2015.1086457
28. Mayol, T.L. (2013). Madagascar's nascent locally managed marine area network. *Madagascar Conservation & Development*, 8(2), 91-95. https://doi.org/10.4314/mcd.v8i2.8

29. Mbaru, E.K., & Barnes, M.L. (2017). Key players in conservation diffusion: Using social network analysis to identify critical injection points. *Biological Conservation*, 210, 222-232. https://doi.org/10.1016/j.biocon.2017.03.031
30. Muehlig-Hofmann, A. (2007). Traditional authority and community leadership: Key factors in community-based marine resource management and conservation. *SPC Traditional Marine Resource Management and Knowledge Information Bulletin*, 21, 31-44. https://www.spc.int/DigitalLibrary/Doc/FAME/InfoBull/TRAD/21/TRAD21_31_Muehlig.pdf
31. Pathak, N. (2006). Community conserved areas in South Asia. *Parks*, 16(1), 56-62.
32. Pero, L.V., & Smith, T.F. (2008). Institutional credibility and leadership: critical challenges for community-based natural resource governance in rural and remote Australia. *Regional Environmental Change*, 8(1), 15-29. https://doi.org/10.1007/s10113-007-0042-4
33. Pomeroy, R.S., Katon, B.M., & Harkes, I. (2001). Conditions affecting the success of fisheries co-management: lessons from Asia. *Marine policy*, 25(3), 197-208. https://doi.org/10.1016/S0308-597X(01)00010-0
34. Ribot, J.C., Lund, J.F., & Treue, T. (2010). Democratic decentralization in sub-Saharan Africa: its contribution to forest management, livelihoods, and enfranchisement. *Environmental Conservation*, 37(1), 35-44. https://doi.org/10.1017/S0376892910000329
35. Rodríguez-Martínez, R.E. (2008). Community involvement in marine protected areas: the case of Puerto Morelos reef, México. *Journal of environmental management*, 88(4), 1151-1160. https://doi.org/10.1016/j.jenvman.2007.06.008
36. Ruiz-Mallén, I. & Corbera, E. (2013). Community-based conservation and traditional ecological knowledge: implications for social-ecological resilience. *Ecology and Society,* 18(4):12. http://dx.doi.org/10.5751/ES-05867-180412
37. Salerno, J., Andersson, K., Bailey, K.M., Hilton, T., Mwaviko, K.K., Simon, I.D., ... & Hartter, J. (2020). More robust local governance suggests positive effects of long-term community conservation. *Conservation Science and Practice*, 3(1), e297. https://doi.org/10.1111/csp2.297
38. Sandström, A., Crona, B., & Bodin, Ö. (2014). Legitimacy in co-management: The impact of preexisting structures, social networks and governance strategies. *Environmental Policy and Governance*, 24(1), 60-76. https://doi.org/10.1002/eet.1633
39. Seixas, C.S., & Davy, B. (2007). Self-organization in integrated conservation and development initiatives. *International Journal of the Commons*, *2*(1), 99-125. DOI:
40. Seixas, C.S., & Berkes, F. (2009). Community-based enterprises: The significance of partnerships and institutional linkages. *International Journal of the Commons*, 4(1), 183–212. http://doi.org/10.18352/ijc.133
41. Shukla, S.R., & Sinclair, A.J. (2010). Strategies for self-organization: learning from a village-level community-based conservation initiative in India. *Human Ecology*, 38(2), 205-215. https://doi.org/10.1007/s10745-010-9301-y
42. Sowman, M., Hauck, M., & Branch, G. (2003). *Lessons learned from nine coastal and fisheries co-management case studies.* In: Hauck, M., Sowman, M. (Eds.), *Waves of Change: Coastal and Fisheries Co-management in South Africa*. Cape Town: Juta and Company, pp. 299–340.

43. Steenbergen, D.J. (2016). Strategic customary village leadership in the context of marine conservation and development in Southeast Maluku, Indonesia. *Human Ecology*, 44(3), 311-327. https://doi.org/10.1007/s10745-016-9829-6
44. Steenbergen, D.J., & Warren, C. (2018). Implementing strategies to overcome social-ecological traps: the role of community brokers and institutional bricolage in a locally managed marine area. *Ecology and Society*, 23(3), 10. https://doi.org/10.5751/ES-10256-230310
45. Sudtongkong, C., & Webb, E.L. (2008). Outcomes of state- vs. community-based mangrove management in southern Thailand. *Ecology and Society*, 13(2), 27. http://www.ecologyandsociety.org/vol13/iss2/art27/
46. Sutton, A.M., & Rudd, M.A. (2014). Deciphering contextual influences on local leadership in community-based fisheries management. *Marine Policy*, 50, 261-269. Dhttps://doi.org/10.1016/j.marpol.2014.07.014
47. Sutton, A.M., & Rudd, M.A. (2016). Factors influencing community fishers' leadership engagement in international small-scale fisheries. *Frontiers in Marine Science*, 3, 116. https://doi.org/10.3389/fmars.2016.00116
48. Tang, C.P., & Tang, S.Y. (2010). Institutional adaptation and community-based conservation of natural resources: the cases of the Tao and Atayal in Taiwan. *Human Ecology*, 38(1), 101-111. https://doi.org/10.1007/s10745-009-9292-8
49. Timmer, V. (2004). *Community-based conservation and leadership: Frameworks for analyzing the equator initiative*. CID Graduate Student Working Paper Series 2004.2. Science, Environment and Development Group, Center for International Development, Harvard University. https://nrs.harvard.edu/URN-3:HUL.INSTREPOS:37366424
50. Ullah, I., & Kim, D.Y. (2020). A model of collaborative governance for community-based trophy-hunting programs in developing countries. *Perspectives in Ecology and Conservation,* 18(3), 145-160. https://doi.org/10.1016/j.pecon.2020.06.004
51. Vaughan, M.B., & Caldwell, M.R. (2015). Hana Pa'a: Challenges and lessons for early phases of co-management. *Marine Policy*, 62, 51-62. http://dx.doi.org/10.1016/j.marpol.2015.07.005
52. Velez, M., Adlerstein, S., & Wondolleck, J. (2014). Fishers' perceptions, facilitating factors and challenges of community-based no-take zones in the Sian Ka'an Biosphere Reserve, Quintana Roo, Mexico. *Marine Policy*, 45, 171-181. http://dx.doi.org/10.1016/j.marpol.2013.12.003
53. Walters, G., Broome, N.P., Cracco, M., Dash, T., Dudley, N., Elías, S., ... & Van Vliet, N. (2021). COVID-19, Indigenous peoples, local communities and natural resource governance. *PARKS*, 47-62.
54. Warren, C., & Visser, L. (2016). The local turn: an introductory essay revisiting leadership, elite capture and good governance in Indonesian conservation and development programs. *Human Ecology*, 44(3), 277-286. https://doi.org/10.1007/s10745-016-9831-z
