# Supplementary material for: Identifying and developing effective post‐2020 conservation bridging leaders
Source: Conserv Biol. 2022 Oct 6;36(6):e13980. doi: 10.1111/cobi.13980 (PMC10092307; doi:10.1111/cobi.13980)
Supplement: Supplementary file 4 — Appendix S4: Social Relations and Network Appraisal Questionnaire, and Interview Guides [file COBI-36-0-s002.pdf]

Appendix S4: Social Relations and Network Appraisal Questionnaire, and Interview Guides

Social Relations and Network Appraisal: Urok Community-Managed Marine Protected Area (CMPA), Guinea-Bissau

Name of Village: ________________ Respondent Number: __________________

Social Network Attribute	Question	Response(s)
**Ultimate decision-making power**	Who do you think possesses the ultimate decision-making power associated with the CMPA? List up to 3	1.______________________________<br>2.______________________________<br>3.______________________________
**Social Support**	Whom do you find the most approachable (i.e. you trust the most) to work with over your natural resource use concerns in the CMPA? List up to 3	1.______________________________<br>2.______________________________<br>3.______________________________
	How often do you discuss these concerns with them? (tick for each of the 3 mentioned above)	1. ☐ **once a week** ☐ **once a month** ☐ **once a year** ☐ **when issues arise**<br>2. ☐ **once a week** ☐ **once a month** ☐ **once a year** ☐ **when issues arise**<br>3. ☐ **once a week** ☐ **once a month** ☐ **once a year** ☐ **when issues arise**
**Knowledge Acquisition**	**From whom do you acquire knowledge or information about the following (List up to 3)?**	
	…legal issues & rights about natural resource access & use?	1.______________________________<br>2.______________________________<br>3.______________________________
	…sources of financial support for the CMPA?	1.______________________________<br>2.______________________________<br>3.______________________________
	…sources of non-monetary resources? i.e. fishing gear, boats, etc.…	1.______________________________<br>2.______________________________<br>3.______________________________
	…ecological aspects of conservation? i.e. status of fish & the marine environment, such as decreasing fish stocks	1.______________________________<br>2.______________________________<br>3.______________________________

**Knowledge Diffusion**	**With whom do you share knowledge or information about the following (List up to 3)**?	
	…legal issues & rights about natural resource access & use?	1.__________<br>2.__________<br>3.__________
	…sources of financial support for the CMPA?	1.__________<br>2.__________<br>3.__________
	…sources of non-monetary resources? i.e. fishing gear, boats, etc.…	1.__________<br>2.__________<br>3.__________
	…ecological aspects of conservation? i.e. status of fish & the marine environment, such as decreasing fish stocks	1.__________<br>2.__________<br>3.__________

Interview Guides:

Bay of Ranobe Community Member Interview
Village Name: ______________________
Respondent Number: ________________

1. **Did you live in this area before the community conservation area was established? Yes/No (Circle one).**
2. **From your own experience, please describe the perceptions and attitudes of the community toward this community conservation area.**
3. **In your experience, what have been some of the difficulties experienced in establishing this community conservation area?**
4. **How is the community represented in this community conservation area? And how effectively are community representatives (e.g. village council members) at representing community interests?**
5. **What are the roles and responsibilities of the different actors in this community conservation area, e.g. community members, village representatives, customary authorities, conservation agency/ government ministry, local government, NGO partners like Reef Doctor, etc.…?**
6. **How would you describe the relations between community members and partner organisations? Have these relations changed since the establishment of this community conservation area? If so, how and why?**
7. **How would you describe the relations between community members and the village representatives? Have these relations changed since the establishment of this community conservation area? If so, how and why?**
8. **How would you describe the relations between community members and FIMIHARA representatives? Have these relations changed since the establishment of this community conservation area? If so, how and why?**
9. **Please list any conditions which you have experienced to be challenging/ favourable in participating in the management of this community conservation area.**
10. **What factors/ conditions are important to increase the functioning of this community conservation area?**
11. **Any additional comments from the respondent?**

Bay of Ranobe Local Representative Interview
Village Name: ______________________
Respondent Number: ________________

1. **Did you live in this area before the community conservation area was established? Yes/No (Circle one).**
2. **Please briefly describe your position and responsibilities within your association.**
3. **What led to this community conservation area being established? And how has your association participated in establishing it? What is the purpose of your association?**
4. **What are the roles and responsibilities of the different actors in this community conservation area, e.g. community members, community representatives, traditional authorities, conservation agency/ government ministry, local government, NGO partners like Reef doctor, academic institutions, private sector partners like Hotels and Dive Operators, etc.…?**
5. **How is the community represented? And how effectively are community representatives representing community interests?**
6. **In your opinion, what do you think is the best way of interacting with your community to improve the management of natural resources? Give reasons.**
7. **In your experience, what have been some of the difficulties experienced in establishing this community conservation area?**
8. **From your own experience, please describe the perceptions and attitudes of the community toward this community conservation area.**
9. **How would you describe the relations between community members and the partner organisation(s) of this community conservation area? And how have these relations changed since establishing the community conservation area.**
10. **How would you describe the relations between the community members and your association? And how have these relations changed since the implementation of your community conservation area.**
11. **Please list any conditions which you have experienced to be challenging/ favourable in participating in the management of this community conservation area. What factors/ conditions are important to increase the functionality of this community conservation area?**
12. **What support does your community and your authority need, and from whom, to manage this community conservation area? (e.g. capacity-building; financial capital; legal rights; etc.…)**
13. **Any additional comments from the respondent?**

Partner Organisation Interview (e.g. NGO; government; academic; private sector)
Respondent Number: __________

1. **What led to your organisation's involvement in establishing this community conservation area?How did your role in this community conservation area come about? How did you approach the community? Or how did the community approach you?**
2. **In your experience, what have been some of the difficulties experienced in establishing this community conservation area?**
3. **What are the governance arrangements of this community conservation area and how does your organisation fit in? Who has decision-making powers? How does the local community participate?**
4. **What are the roles and responsibilities of the different actors in this community conservation area, e.g. community members, village representatives, traditional authorities, conservation agency and /or government ministry, local government, NGO partners, academic institutions, private sector partners, etc.…?**
5. **How is the community represented? And how effectively are community representatives representing community interests?**
6. **From your own experience, please describe the attitudes and perceptions of local communities toward this community conservation area.**
7. **How would you describe the relations between community members and your (and other) partner organisation(s) of this community conservation area? And have these relations changed since establishing the community conservation area? If so, how and why?**
8. **How would you describe the relations between the community members and their village representatives? And have these relations changed since establishing the community conservation area? If so, how and why?**
9. **How would you describe the relations between the community members and FIMIHARA? And have these relations changed since establishing the community conservation area? If so, how and why?**
10. **In your opinion, what do you think is the best way of engaging local communities in conservation management activities?**
11. **Please list any conditions which you have experienced to be challenging/ favourable in participating in the management of this community conservation area. And what factors/ conditions are important to increase the functionality of this community conservation area?**
12. **Any additional comments from the respondent?**
