# Supplementary material for: Identifying and developing effective post‐2020 conservation bridging leaders
Source: Conserv Biol. 2022 Oct 6;36(6):e13980. doi: 10.1111/cobi.13980 (PMC10092307; doi:10.1111/cobi.13980)
Supplement: Supplementary file 5 — Appendix S5: Community‐perceived relations with identified bridging leaders [file COBI-36-0-s004.pdf]

Appendix S5: Community-perceived relations with identified bridging leaders

Figures: Community perceptions of identified CBC bridging leaders by percentage of respondents in the Bay of Ranobe, and Urok Islands: overall a) and b); for c) Reef Doctor, and d) Tiniguena; for the respective representatives on e) FIMIHARA, and f) UMC; and for the respective village representatives g) and h). Note: see legends at the end for village codes.

The Bay of Ranobe | The Urok Islands

Perceived relations with identified bridging leaders

a)

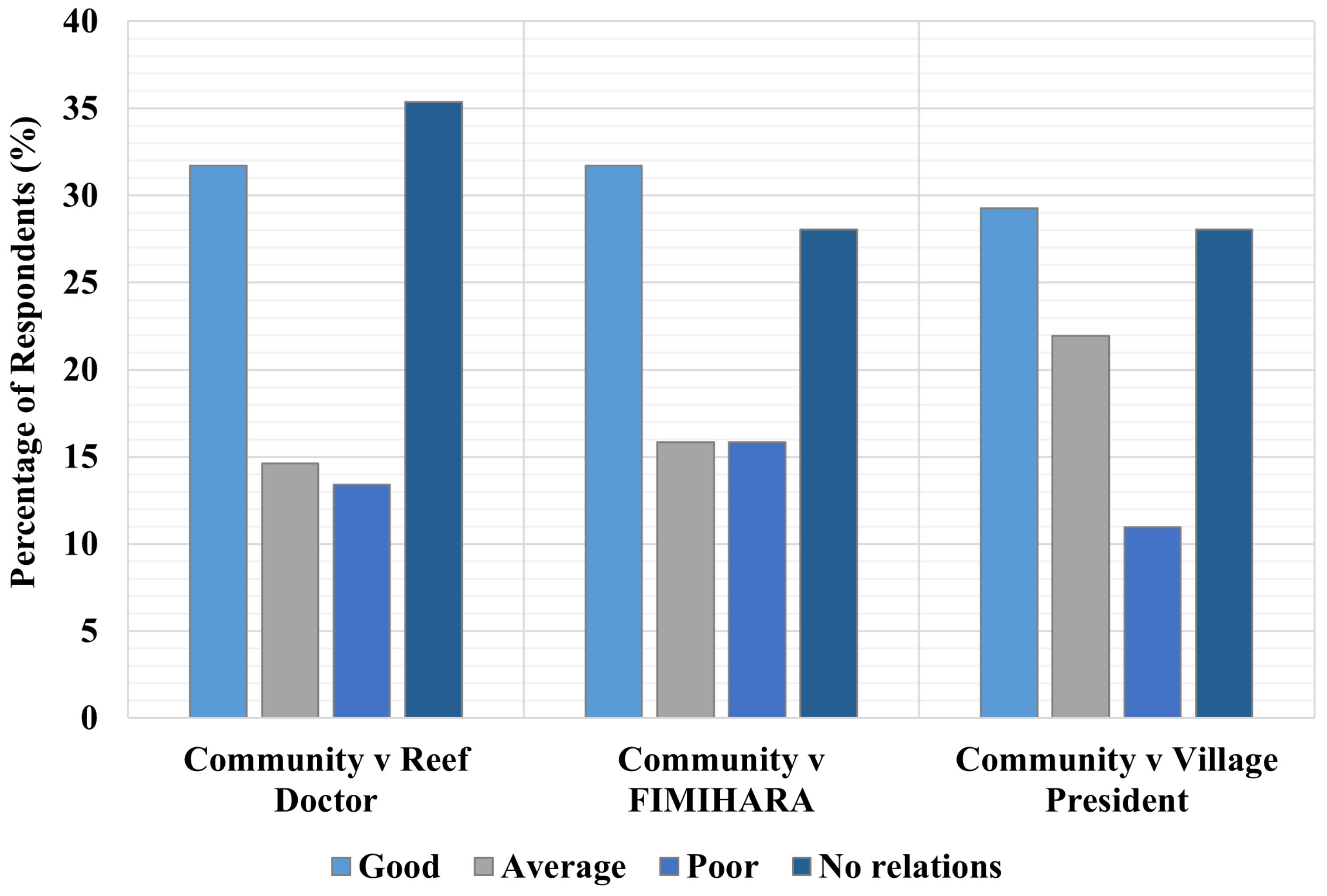

b)

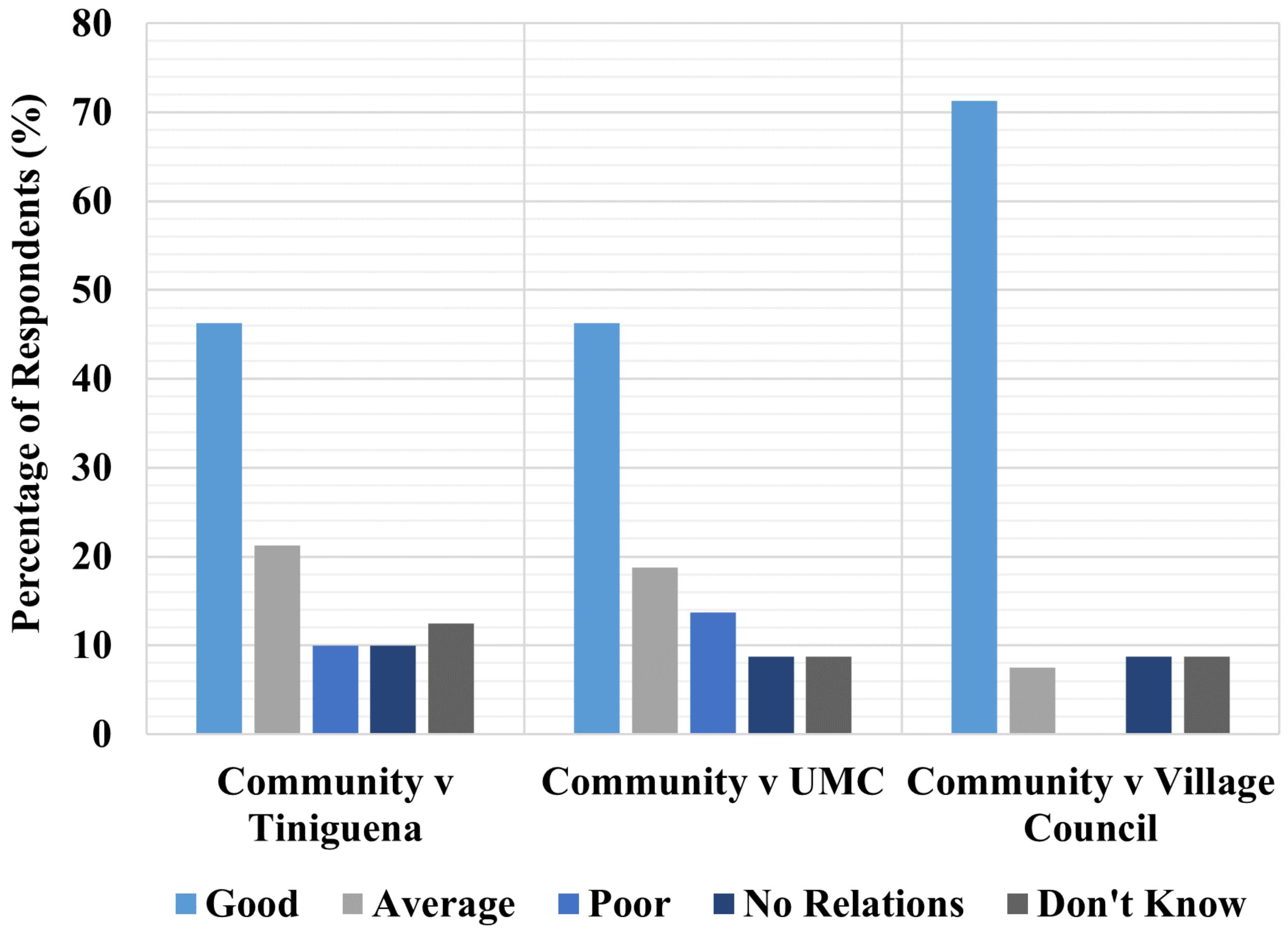

Perceived relations with Reef Doctor/ Tiniguena

c)

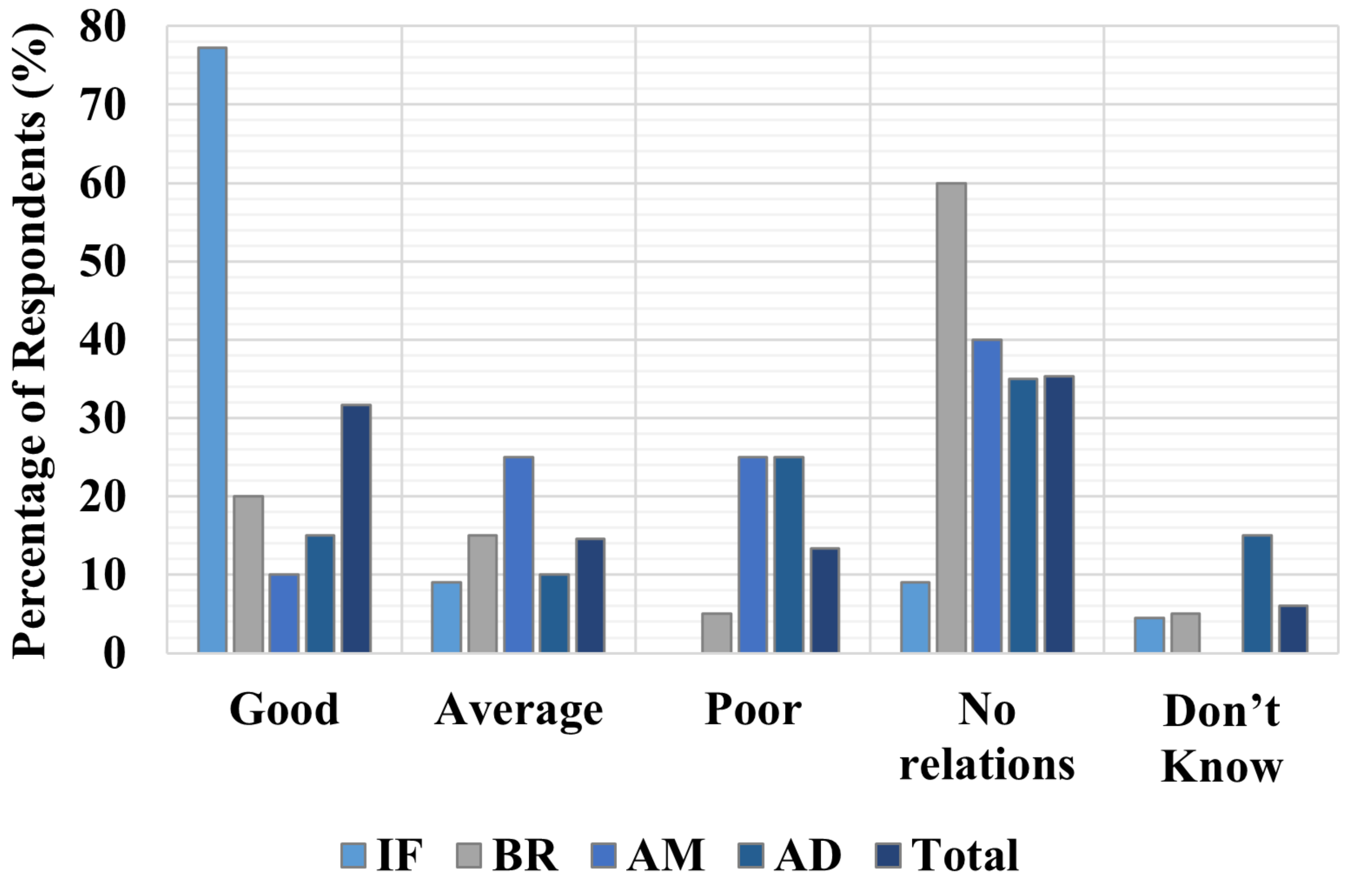

d)

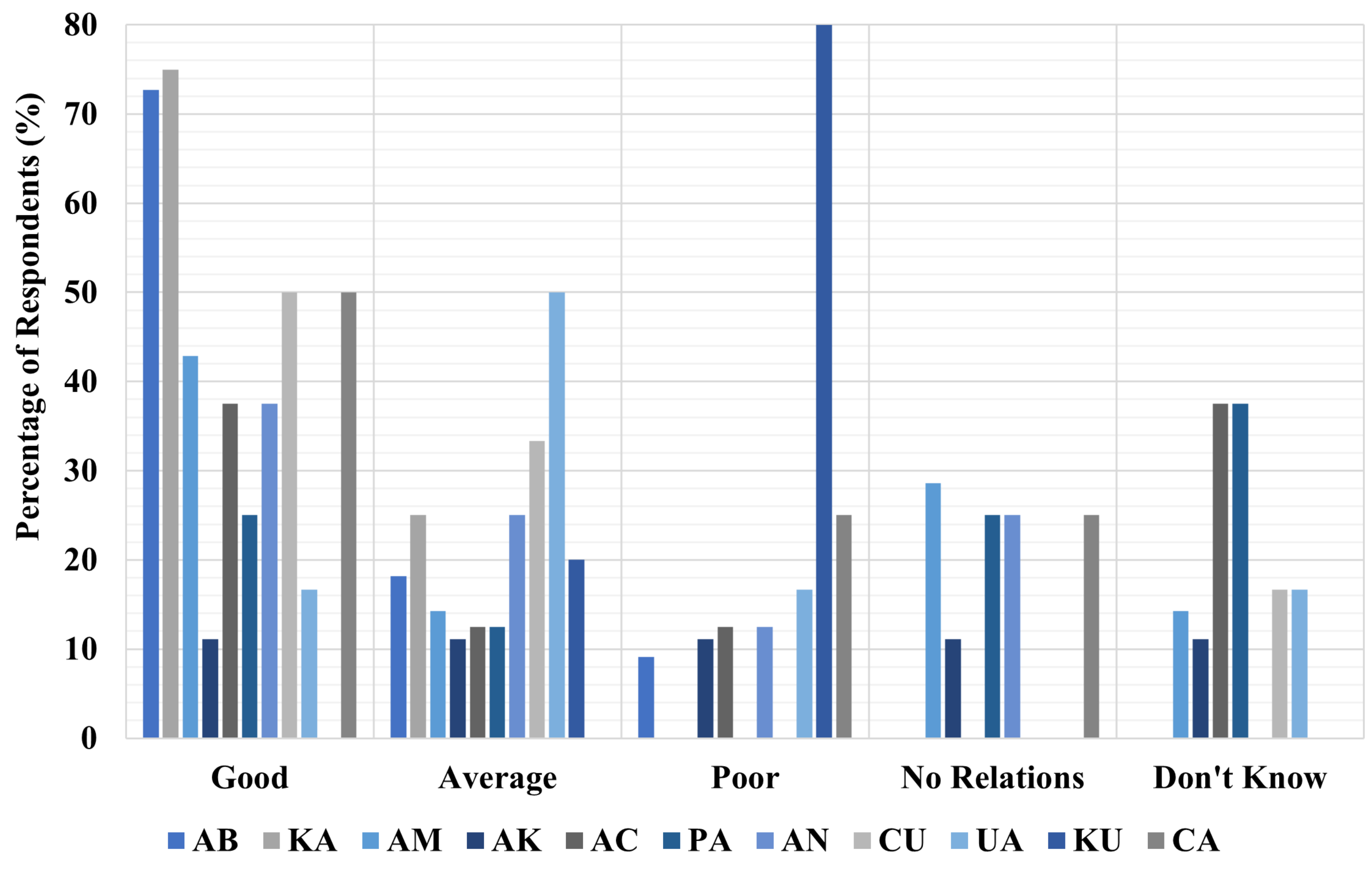

Perceived relations with FIMIHARA/ UMC Representatives

e)

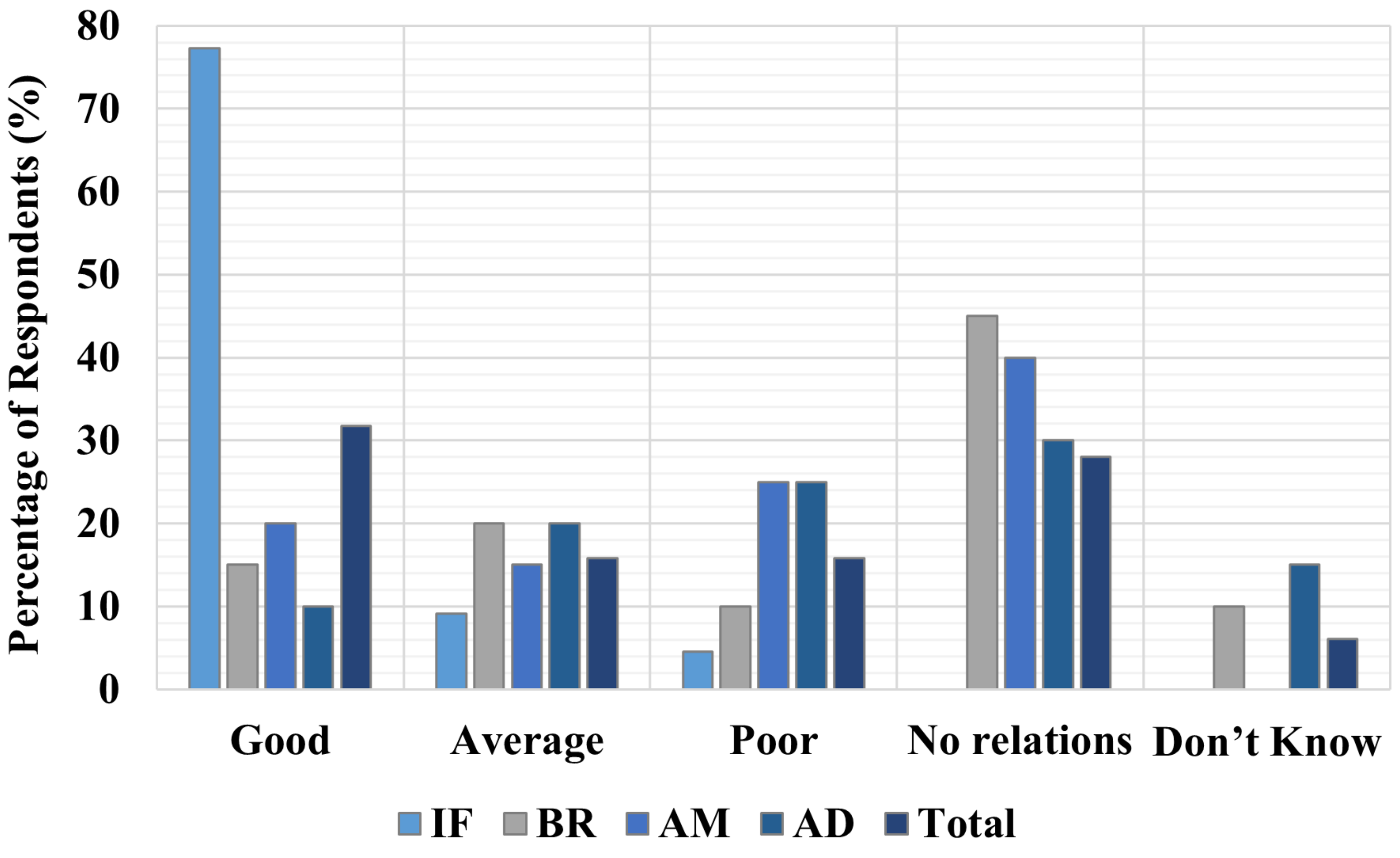

f)

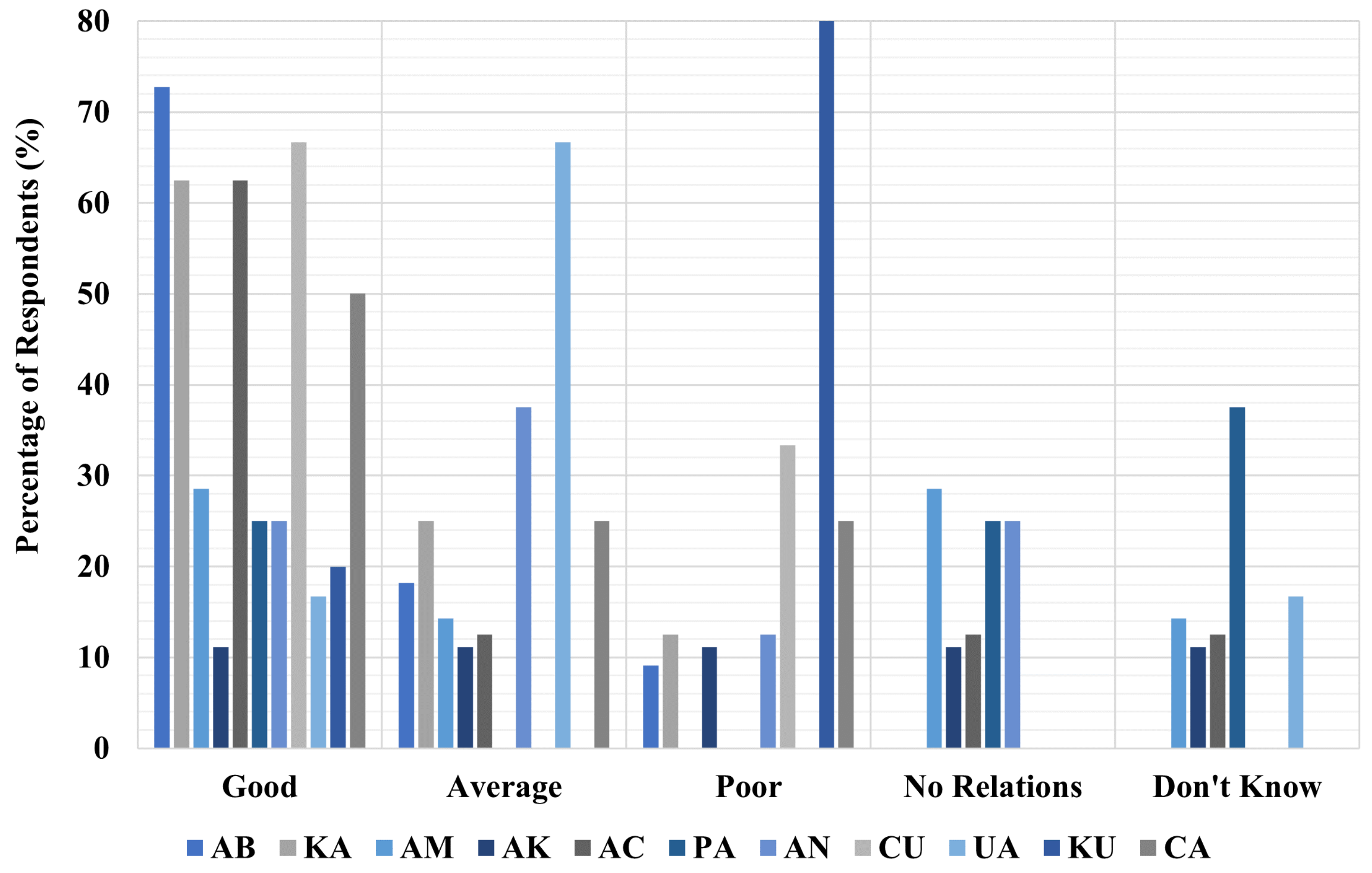

Perceived relations with Village Representatives

g)

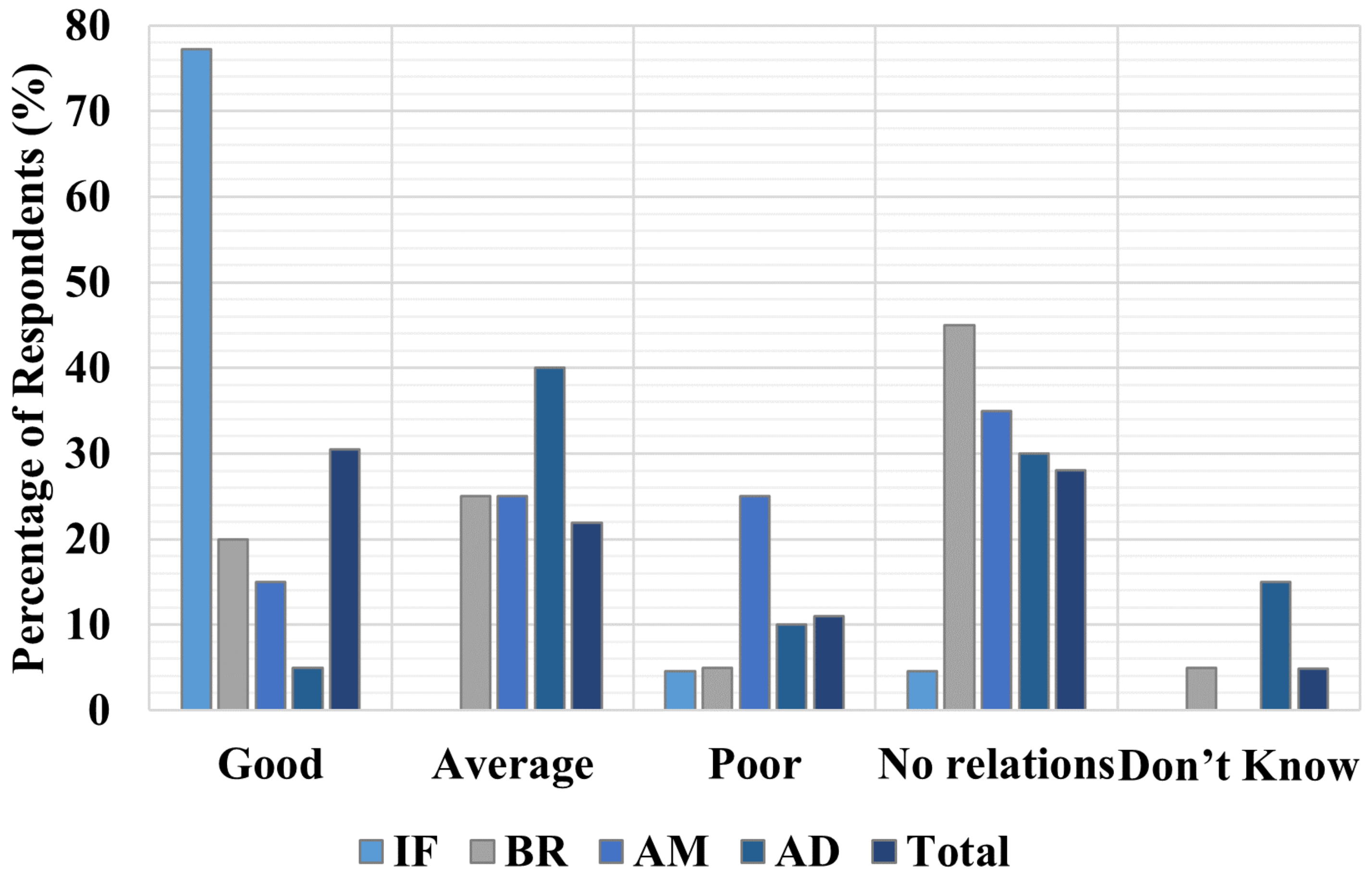

h)

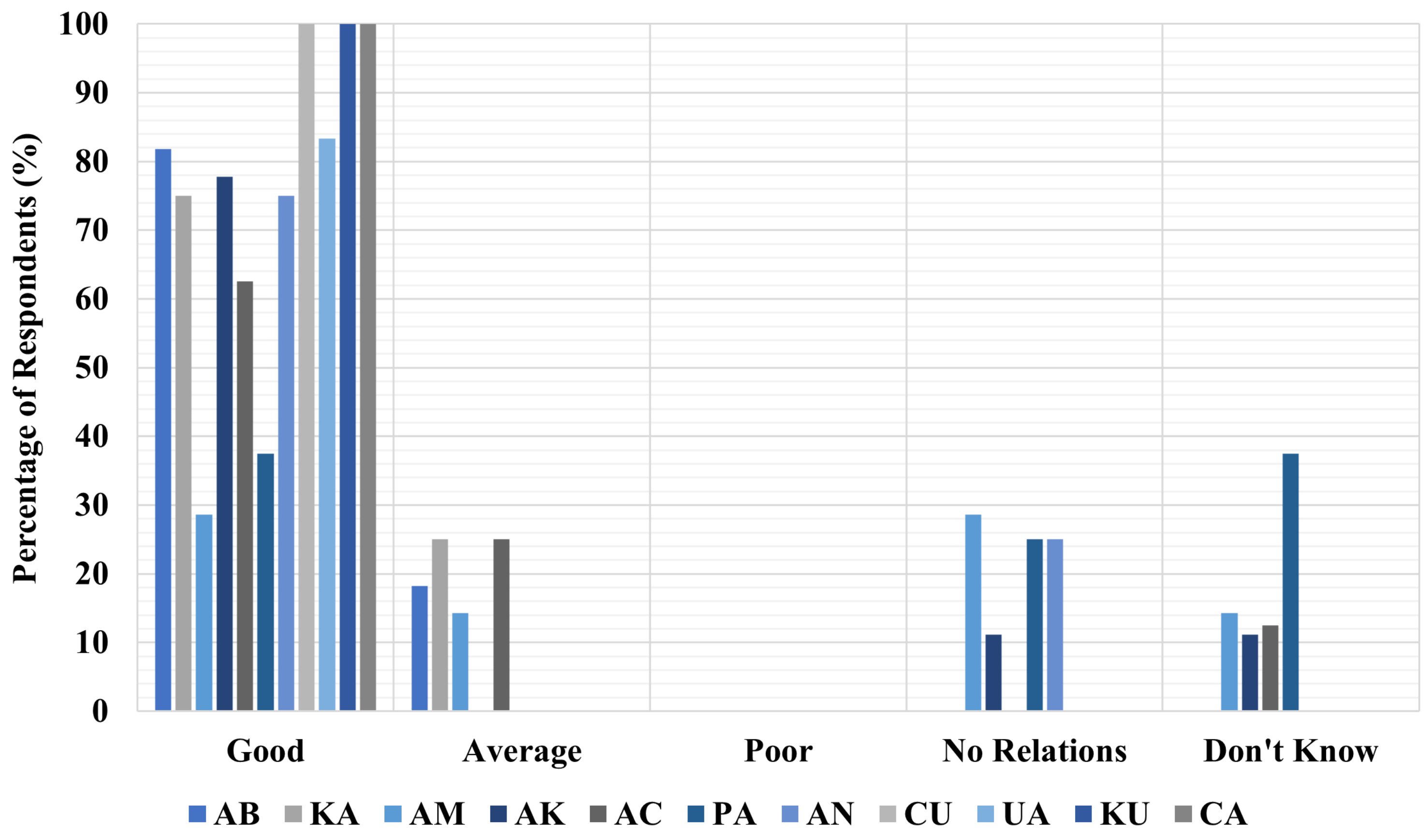

Bay of Ranobe Village Codes:
Ifaty (IF)
Beravy (BR)
Ambolomailaka (AM)
Andrevo (AN)

Urok Islands Village Codes:
Abu (AB)
Kabinhate (KA)
Ambo (AM)
Akoco (AK)
Acuno (AC)
Pandja (PA)
Andamaka (AN)
Cumpada (CU)
Uada (UA)
Kuian (KU)
Caten (CA)
